# Kinetically Controlling Surface Atom Arrangements in Thermally Robust, Amorphous High‐Entropy Alloy Nanoparticles by Solvent Selection

**DOI:** 10.1002/advs.202510537

**Published:** 2025-10-13

**Authors:** Varatharaja Nallathambi, Se‐Ho Kim, Andrea M. Mingers, Petra Ebbinghaus, Baptiste Gault, Sven Reichenberger, Dierk Raabe, Stephan Barcikowski

**Affiliations:** ^1^ Technical Chemistry I and Center for Nanointegration Duisburg‐Essen (CENIDE) University of Duisburg‐Essen 45141 Essen Germany; ^2^ Max Planck Institute for Sustainable Materials Max‐Planck‐Str.1 40237 Düsseldorf Germany; ^3^ Department of Materials Science & Engineering Korea University Seoul 02841 Republic of Korea; ^4^ Department of Materials Royal School of Mines Imperial College London London SW72AZ UK

**Keywords:** amorphous nanoparticles, atom probe tomography, compositionally complex alloys, high‐entropy alloys, in situ electron microscopy, laser ablation, nanoparticle synthesis

## Abstract

The ability to tailor nanoscale surface atom arrangements through multi‐elemental compositional control provides high‐entropy nanoalloys with promising functional properties. Developing a fundamental understanding of nanoalloy formation mechanisms during synthesis is therefore essential for effectively engineering the surface composition and resulting functional properties. Using the Cantor alloy (CrMnFeCoNi) as a model system, the investigation reveals how solvent selection during reactive, nanosecond‐pulsed laser synthesis influences carbon doping and the resulting changes in nanoparticle morphology, structure, and composition. Supersaturated carbon incorporation, partitioned from the organic solvent molecules, produces amorphous nanoparticles with distinctive carbon shells, thermally stable up to 350 °C. Kinetically controlled particle formation mechanisms are proposed, rationalizing the criticality of the time scales between the competing reactions of carbon doping, carbon shell formation, and coalescence of metallic fragments, which rule compositional and morphological characteristics. Carbon shell thickness and the surface composition distribution are shown to influence the element‐specific dissolution under electrochemical reaction conditions. This work demonstrates effective solvent‐driven surface‐compositional control in amorphous high‐entropy nanoalloys. It introduces a novel synthesis approach for tailoring surface atom arrangements through carbon incorporation via reactive, pulsed laser synthesis.

## Graphical abstract

This work leverages the level of carbon supersaturation in the metallic constituents during particle formation to regulate nanoparticle morphology, structure, and composition distribution. The solvent‐dependent (C/(C+O)) ratio and the relative kinetics of the competing reactions of carbon shell formation, coalescence of metallic constituents, and particle growth determine the nanoparticle characteristics as evidenced in three different solvents.

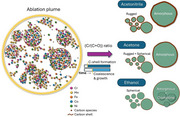

## 1 Introduction

Nanomaterials with their unique size and shape effects are attractive for a wide range of applications, including electronics,^[^ ^1^ ^]^ optoelectronics,^[^ ^2^ ^]^ catalysis,^[^ ^3^, ^4^, ^5^ ^]^ biomedicine,^[^ ^6^ ^]^ energy conversion and storage.^[^ ^7^ ^]^ Doping or alloying nanoparticles with solute elements is a well‐established strategy to improve their functional properties.^[^ ^8^, ^9^, ^10^ ^]^ Extending this concept to high‐entropy alloys (HEA) or compositionally complex alloys (CCA), with multiple principal elements mixed in near‐equiatomic proportions that form a single‐phase solid solution, is a new frontier in heterogeneous catalysis^[^ ^11^, ^12^, ^13^, ^14^, ^15^ ^]^ and in developing more efficient nanomaterials for high‐temperature applications.^[^ ^16^ ^]^ The concept of HEAs originally focused on solid solutions stabilized by enhanced configurational entropy, and now the term broadly includes compositionally complex materials with extended solid solution ranges, not limited to those stabilized solely by entropy.^[^ ^12^, ^17^ ^]^ To maintain similarity with the literature on this topic, we proceed to use the term HEA in the present work.

In bulk, HEAs have been explored for their promising mechanical, magnetic, and high‐temperature behavior, and the ability to adjust composition and atomic configurations in these materials opens a practically infinite chemical and structural space for designing multifunctional nanoalloys.^[^ ^13^, ^18^, ^19^, ^20^ ^]^ The complex multi‐elemental surface atom arrangements can induce unprecedented electronic and optical responses that open opportunities for optimizing the functional performance, for example, in catalyzing specific chemical reactions.^[^ ^12^, ^21^ ^]^

Achieving rational and controllable synthesis with precise control over the microstructure, morphology, and composition of HEAs remains a significant challenge,^[^ ^22^, ^23^, ^24^ ^]^ particularly for nanomaterials with a high surface‐to‐volume ratio needed for catalytic activities.^[^ ^17^, ^25^, ^26^ ^]^ The vast compositional space available to design HEA catalysts also faces limitations because of miscibility gaps and the contribution of the positive enthalpy of mixing between the constitutive binary and ternary systems involved, leading to phase separation.^[^ ^27^, ^28^ ^]^ In addition, the random mixing of the elemental components in nanostructures is difficult to achieve as most approaches require high temperatures, potentially favoring demixing, reacting, or stabilizing agents that can alter the chemistry, or controlled environments that complicate upscaling and control of the synthesis.

Laser synthesis and processing of colloids (LSPC) is a well‐established and robust synthesis technique with the unique advantage of producing surfactant‐free, colloidally‐stable nanoparticles (NPs) with exceptional compositional freedom and high productivity.^[^ ^29^, ^30^, ^31^, ^32^, ^33^ ^]^ LSPC encompasses a variety of techniques, namely laser ablation, fragmentation, melting, and reduction in liquids, and offers vast flexibility in the type, form, and composition of the source material and the solvent medium used.^[^ ^34^ ^]^ In addition, the rapid heating and fast cooling rates during NP generation help the formation of thermodynamically metastable nanostructures, kinetically hindering the equilibration process.^[^ ^35^, ^36^ ^]^ Recent studies reported the synthesis of HEA NPs using laser ablation in liquids^[^ ^21^, ^37^, ^38^, ^39^, ^40^, ^41^ ^]^ as well as laser reduction of metal precursor mixtures.^[^ ^42^ ^]^ The solvent used during the synthesis influences the nature of the reactive species formed from molecular decomposition during pulsed laser irradiation.^[^ ^43^, ^44^ ^]^ In organic solvents, the reactive carbon species released in situ promote chemical reactions with the forming nanostructures, altering stoichiometry and creating NPs surrounded by carbon shells.^[^ ^44^, ^45^, ^46^, ^47^ ^]^ The formation of amorphous CrMnFeCoNi and CrMnFeCoNiMo NPs, in addition to minor fractions of the fcc phase, through LSPC in acetonitrile has been previously reported.^[^ ^39^ ^]^ A significant carbon doping in the NP volume and the confinement effect created by the carbon shell were hypothesized to stabilize the amorphous structure. However, a mechanistic understanding of the particle formation mechanisms and solvent‐metal interactions influencing the characteristics of the generated amorphous HEA NPs is missing.

Therefore, this study deploys a multi‐microscopy and spectroscopy approach to report on the structure and morphology of the carbon‐containing CrMnFeCoNiC_x_ HEA nanoparticles, along with their size‐dependent composition distribution and thermal stability. We investigate the synthesis and in situ carbon doping of HEA NPs by reactive nanosecond‐pulsed laser ablation in acetonitrile, ethanol, and acetone. This allows us to report on the systematic influence of the solvent‐metal interactions during laser synthesis on the resulting characteristics of the HEA NPs using transmission electron microscopy (TEM) combined with selected area electron diffraction (SAED), scanning TEM (STEM) paired with energy dispersive X‐ray spectroscopy (EDS), atom probe tomography (APT) and X‐ray photoelectron spectroscopy (XPS). The produced particles are found to be amorphous with different degrees of carbon shell thickness depending on the solvent used. In situ TEM heating studies demonstrate that carbon supersaturation is the rationale behind the amorphous phase formation, as it kinetically hinders crystallization during particle formation. A mechanistic view of the particle formation during reactive laser ablation is proposed, and the impact of the relative kinetics of competing reactions on the formation of carbon shells and the compositional distribution is discussed. Element dissolution profiles obtained via scanning flow cell measurements with inductively coupled plasma–mass spectrometry (SFC–ICP–MS) demonstrate the influence of carbon shell thickness and surface composition on element‐specific dissolution under applied potential conditions. Our results evidence a solvent‐driven structural and compositional control of amorphous, carbon‐doped HEA nanoparticles, with LSPC proving its potential for the synthesis of compositionally complex nanostructures in high volume.

## 2 Results and Discussion

### 2.1 Characteristics of Nanoparticles Synthesized in Acetonitrile

**Figure 1:**
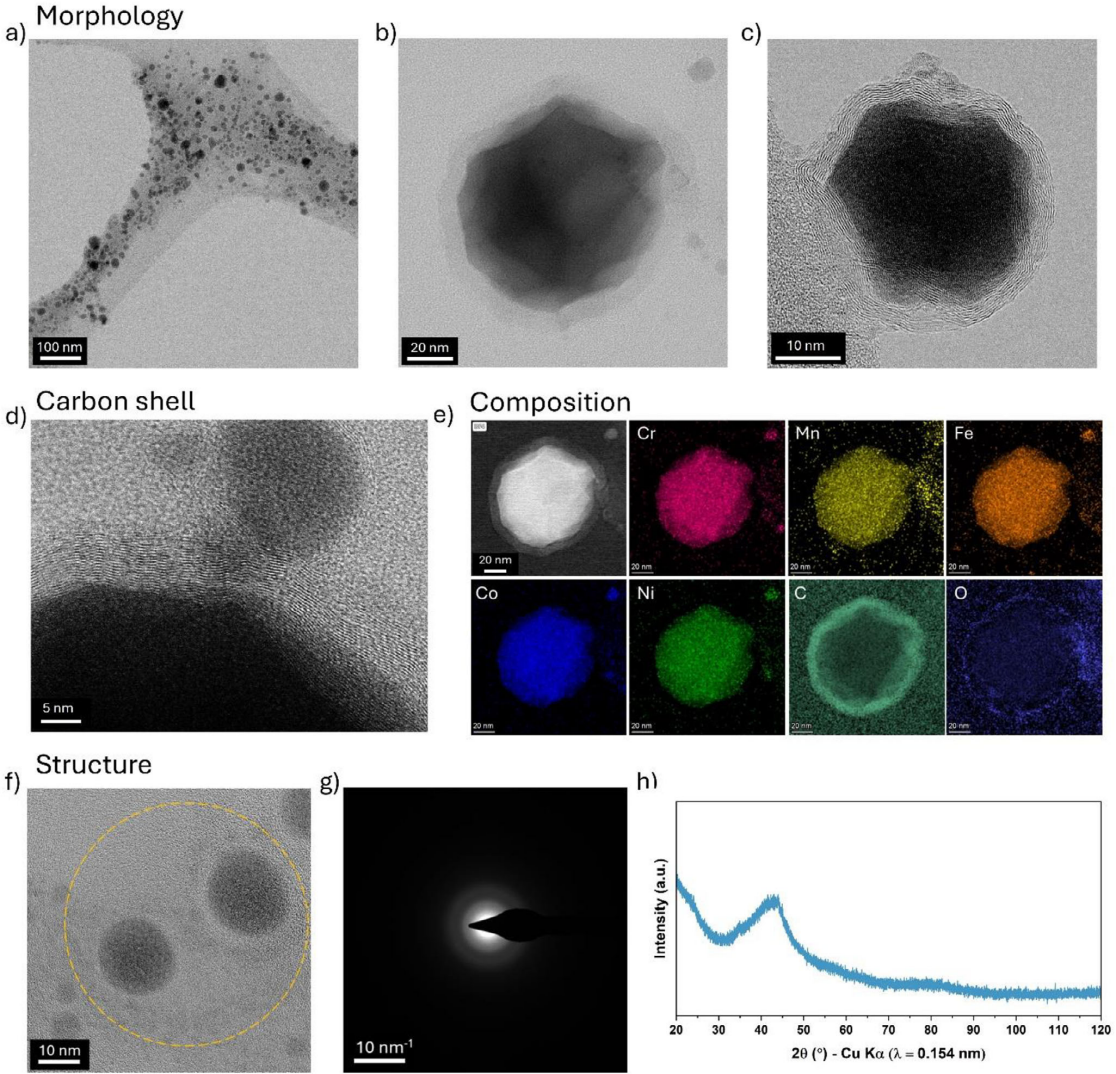
Morphology, composition, and structural characterization of HEA NPs synthesized in acetonitrile. a) TEM bright field image showing the nanoparticles' morphology and size variations; b,c) STEM bright field images of selected nanoparticles of different sizes highlighting their rugged surface covered with a carbon shell around; d) STEM bright field image at a higher magnification showing the onion‐like morphology and size‐dependent carbon shell thickness differences between two NPs; e) STEM‐EDS analysis showing the individual element distribution maps; f,g) TEM bright field image and its respective SAED pattern highlighting the amorphous nature of the NPs; h) Powder XRD pattern of the NPs showing a diffused peak indicating their amorphous nature.

The colloidal HEA NPs synthesized by LSPC in acetonitrile were imaged by TEM (**Figure** **1**a). Details of the structural and compositional characterization of the target material are discussed in the Supporting Information. The particle size ranges from 5 nm to hundreds of nanometers with an average diameter of 17 ± 5 nm (Figure S1, Supporting Information). The HEA NPs synthesized in acetonitrile exhibit an irregularly shaped, rugged morphology (Figure 1b,c), which contrasts with the spherical particles obtained through conventional laser ablation synthesis, suggesting distinct particle formation and growth mechanisms. Each particle is encapsulated by a graphitic, onion‐like carbon shell (Figure 1d). The graphitic nature of the carbon shells is further evidenced by the observation of G and D bands in Raman spectroscopy (Figure S3, Supporting Information). Mapping by STEM‐EDS reveals a near‐uniform distribution of constituent metallic elements, Figure 1e. Carbon is predominantly in the outer shell but is found throughout the particle volume (Figure S2, Supporting Information). The diffuse rings in the SAED pattern, Figure 1f and g, indicate amorphous NPs (Figures S4–S7, Supporting Information), further confirmed by XRD of the dried colloid, Figure 1h, with a single broad peak at 2θ = 43°.

**Figure 2:**
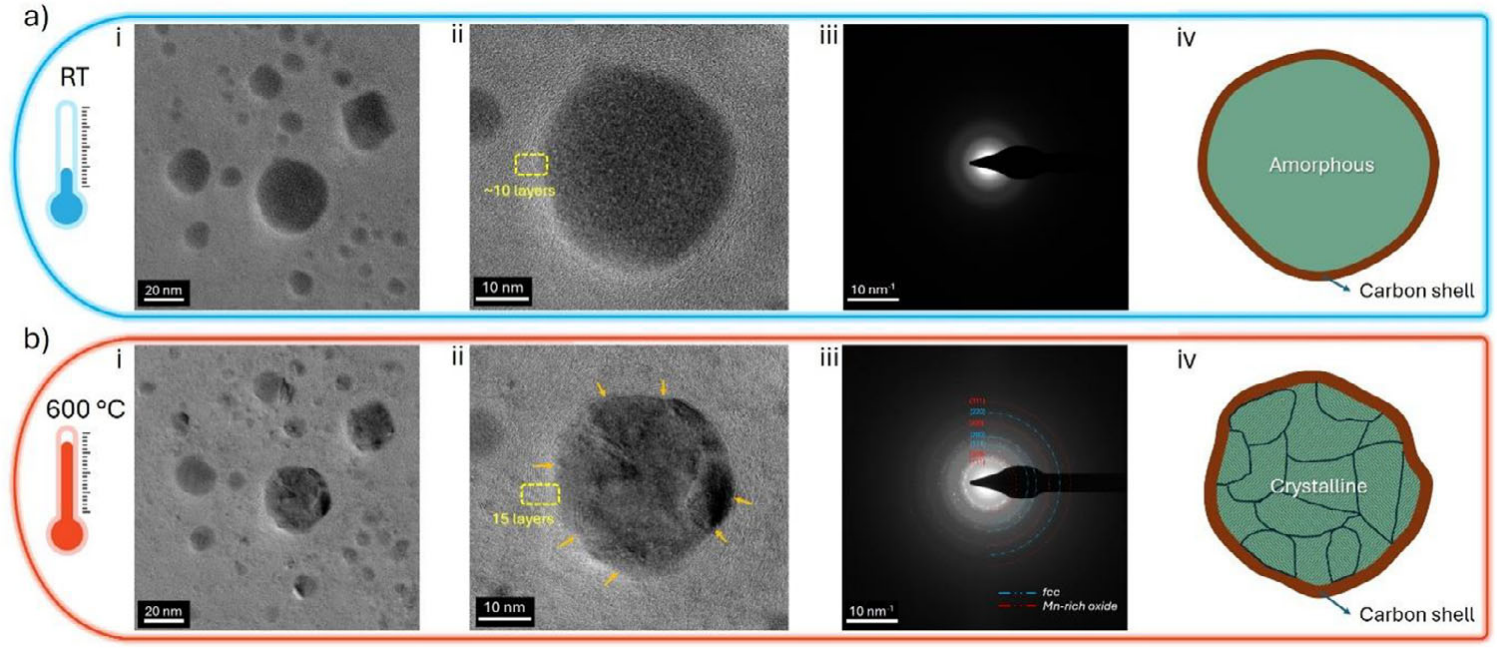
In situ TEM heating studies of HEA NPs synthesized in acetonitrile. a) TEM characterization of as‐synthesized NPs: i) TEM bright field image of a selected NPs region showing their amorphous state up to 350 °C (see Figure S3, Supporting Information), ii) TEM bright field image highlighting the carbon shell morphology around a selected NP, iii) SAED pattern of the NP shown in (ii) highlighting its amorphous nature, and iv) a schematic representation of the NP in its as‐synthesized condition. b) TEM characterization of in situ heated NPs at 600 °C: i) TEM bright field image of a selected NPs region showing their transition to a crystalline state, ii) TEM bright field image highlighting the evolution of carbon shell thickness around a selected NP, iii) SAED pattern of the NP shown in (ii) highlighting its crystalline nature after heating to 600 °C and iv) a schematic representation of the evolution of the NP after heating to 600 °C.

The thermal stability and microstructural evolution of these NPs were evaluated by in situ TEM (see Methods), and **Figure** **2**a,b shows variations observed before and after heating up to 600 °C. Crystallization started in smaller particles (<10 nm in diameter) during the heating step of 350 to 400 °C (Figure S3, Supporting Information). At higher temperatures, crystallites nucleate in the larger particles and then grow till 600 °C, evidenced by the enhanced contrast, Figure 2b‐i and iii. Rings in the SAED before and after heating, Figure 2a‐iii and b‐iii, evidence polycrystalline, face‐centered cubic particles, along with a Mn‐rich oxide phase. During heating, the onion‐like graphitic carbon shell thickness increased from ≈10 to ≈15 layers after reaching 600 °C, driven by the outward diffusion of carbon from the bulk of the NP toward the shell. The equilibrium solubility of carbon in the lattice decreases due to crystallization and segregates to the surface. From thermodynamic modeling, the equilibrium C solubility in the face‐centered cubic phase of CrMnFeCoNi alloy reported is relatively low at ≈0.1 at.% at 1000 °C.^[^ ^48^ ^]^ Sharp edges of crystallites marked by yellow arrows in Figure 2b‐ii indicate that the exclusion of carbon is essential to enable crystallization, at the expense of increasing the surface energy, highlighting the critical role of carbon supersaturation^[^ ^44^, ^49^ ^]^ in pulsed laser synthesis in organic solvents.

### 2.2 Size‐Dependent Composition Distributions of Nanoparticles in Acetonitrile

**Figure 3:**
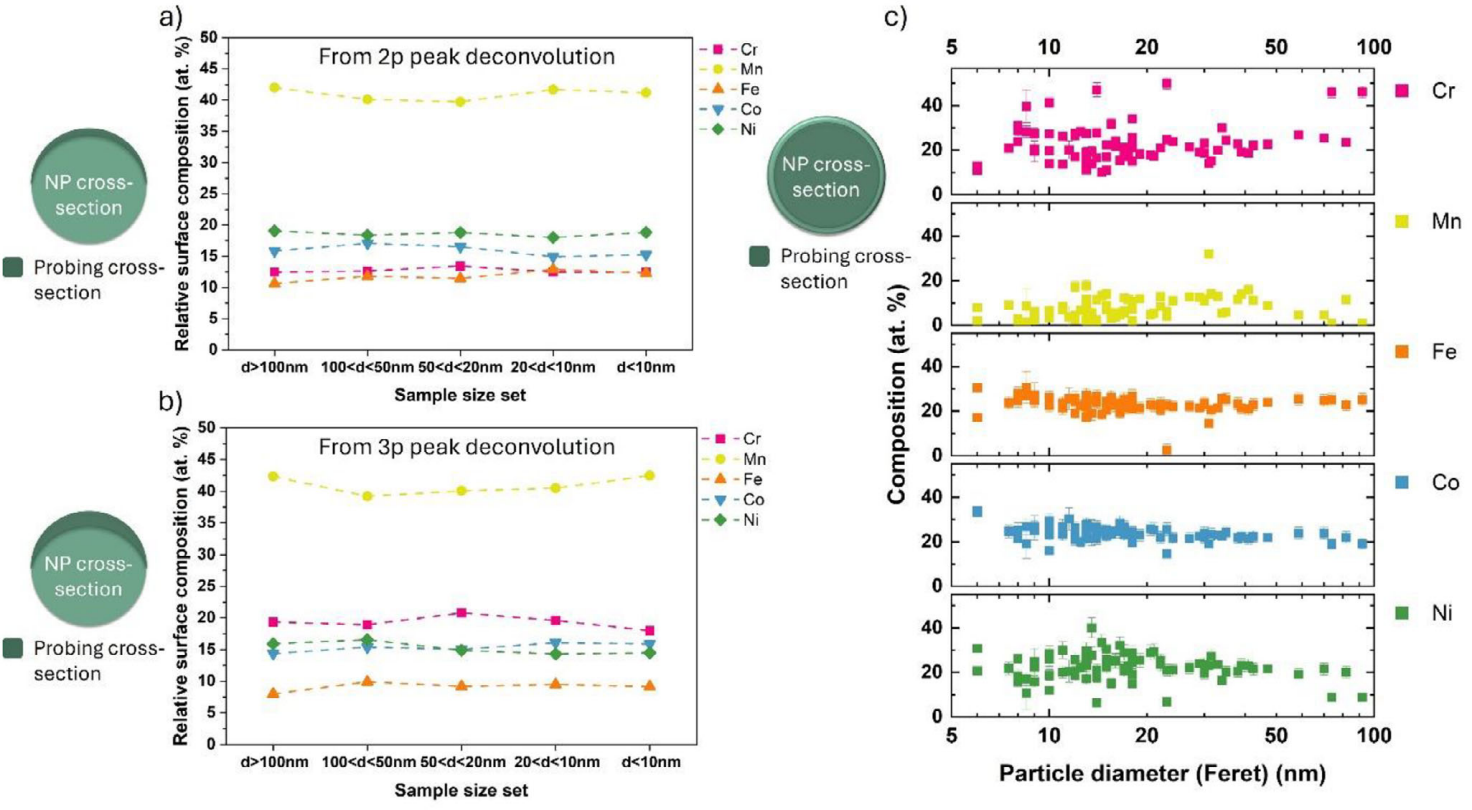
Size‐dependent composition distributions in HEA NPs synthesized in acetonitrile. a,b) Relative XPS surface elemental compositions as a function of different particle diameter sub‐sets calculated from the high‐resolution XPS 2p and 3p spectra of the respective elements. c) Average elemental compositions as a function of particle diameter obtained from STEM‐EDS. The schematic representation on the left of the plots shows relative probing volume cross‐sections compared to the NP cross‐sections of the respective measurement variant.

By centrifugation, the as‐synthesized amorphous HEA NPs of different sizes were separated into five classes (see Experimental Section). Quantitative analysis of XPS spectra gives the relative composition integrated over a few nanometers below the particle's surface, ≈4 and ≈6–7 nm for 2p and 3p signals, respectively^[^ ^50^, ^51^, ^52^ ^]^ (see Experimental Section). The surface composition is consistently offset from the equimolar composition of the target (**Figure** **3**a,b). The concentration of Mn exceeds 40 at.%, whereas that of Fe is only 7–10 at.%, and Co and Ni are 13–17 at.%. The concentration of Cr is ≈13 at.% from deconvoluting the 2p signals and 20 at.% for 3p, indicating Cr segregation beneath a Mn‐enriched surface. These measurements agree with previous computational studies on bulk crystalline Cantor alloys.^[^ ^53^, ^54^, ^55^ ^]^ Transition metals, particularly Mn and Cr, are prone to oxidation in the conditions of laser synthesis,^[^ ^41^ ^]^ which involve supercritical vapor and liquid phases of the metallic constituents along with active species like carbon, oxygen, and hydrogen in the vicinity of the ablation plume.^[^ ^29^ ^]^ Oxidation is expected to be minimized in organic solvents compared to aqueous media, yet it can drive a deviation from stoichiometry in the condensed NPs. STEM‐EDS mapping (Figure 1e; Figure S2, Supporting Information) of individual NPs shows little to no detectable oxygen within the particle volume. Hence, the Mn‐enriched surface cannot be attributed to oxidation during ablation. A shift of the peaks in the XPS spectra from the metallic species (+3.1 eV for Cr 2p_3/2_, +2.3 eV for Mn 2p_3/2_, +2.2 eV for Fe 2p_3/2_, +3.8 eV for Co 2p_3/2_, and +3.6 eV for Ni 2p_3/2_) indicates the presence of oxide/hydroxides from possible oxidation post‐ablation.

Figure 3c plots the composition from STEM‐EDS, averaged through the thickness of the particle, as a function of particle diameter (Feret). Mn depletion, typically above 10 at.%, is evidenced regardless of size. Control measurements were made to eliminate deviations from specimen preparation or plasma cleaning of the grids prior to analysis (see Experimental Section). Fe and Co concentrations remain near‐stoichiometric. Cr and Ni concentrations show considerable scatter, particularly in NPs below 20 nm, while approaching stoichiometric values in larger particle sizes. The Cr enrichment observed in nanoparticles ≈20 nm can be attributed to its pronounced sub‐surface enrichment tendency, whereas Ni exhibits the opposite trend, as also evidenced in the XPS composition profiles. STEM‐EDS mappings, Figures S9–S13 (Supporting Information), show Mn‐enriched fragments outside the NP regions and attached to the carbon shells, which contribute to surface Mn‐enrichment in XPS. The Mn loss hence occurred during the ablation process, before the formation of the carbon shells, and the co‐location of Mn and O in Figure 1e suggests the Mn‐fragments formed oxides, likely during storage.

**Figure 4:**
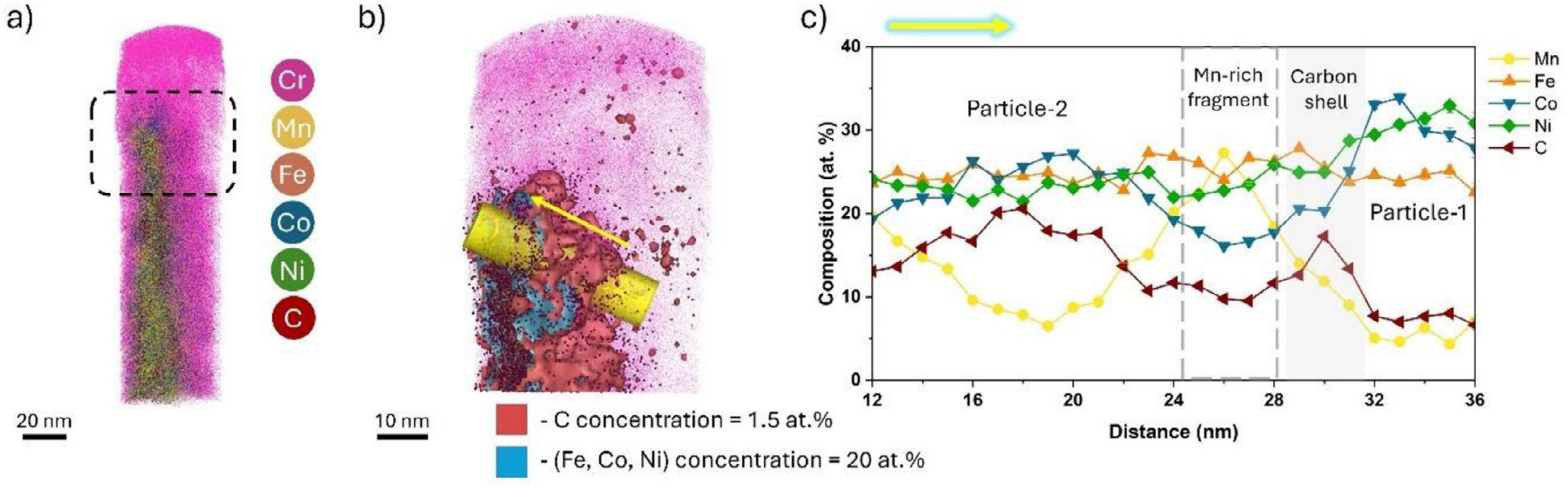
APT analysis of HEA NPs synthesized in acetonitrile. a) 3D APT reconstruction of a NP assembly encapsulated in an in situ coated Cr layer; b) Atom map from a selected region highlighting the carbon shell surrounding the NP region using an isosurface (brown) of C with a concentration >1.5 at.%; c) 1D composition profile from the selected ROI shown in (b) highlighting the composition distribution as a function of distance.

Complementary compositional analysis was obtained by APT, performed on an NP assembly and facilitated by in situ Cr‐coating,^[^ ^56^ ^]^ with the corresponding 3D reconstruction displayed in **Figure** **4**a. The use of Cr for the coating prevents us from analyzing the distribution of Cr in the NPs themselves. Figure 4b is a close‐up on the carbon shell surrounding the NPs highlighted by a brown isosurface, delineating regions containing >1.5 at.% C, surrounding NPs mapped by a blue isosurface delineating concentration >20 at.% of Fe, Co, and Ni combined. The complex elemental environment with substantial C content (up to 20 at.%) in these NPs can induce local magnification and trajectory aberrations due to non‐uniform evaporation fields, complicating the delineation of individual NPs.^[^ ^57^, ^58^, ^59^ ^]^ A 1D composition profile calculated along a cylindrical region of interest (ROI – yellow), plotted in Figure 4c, provides the composition distribution between two NPs. A range of 12–15 at.% of C is found within the NPs, and while Ni, Co, and Fe are relatively uniformly distributed, Mn is depleted. A carbon‐enriched shell appears between the two particles, and the peak in Mn concentration is interpreted as Mn‐rich fragments on the surface, as otherwise observed by STEM‐EDS and XPS.

### 2.3 Insights into Amorphous Nanoparticle Formation

**Figure 5:**
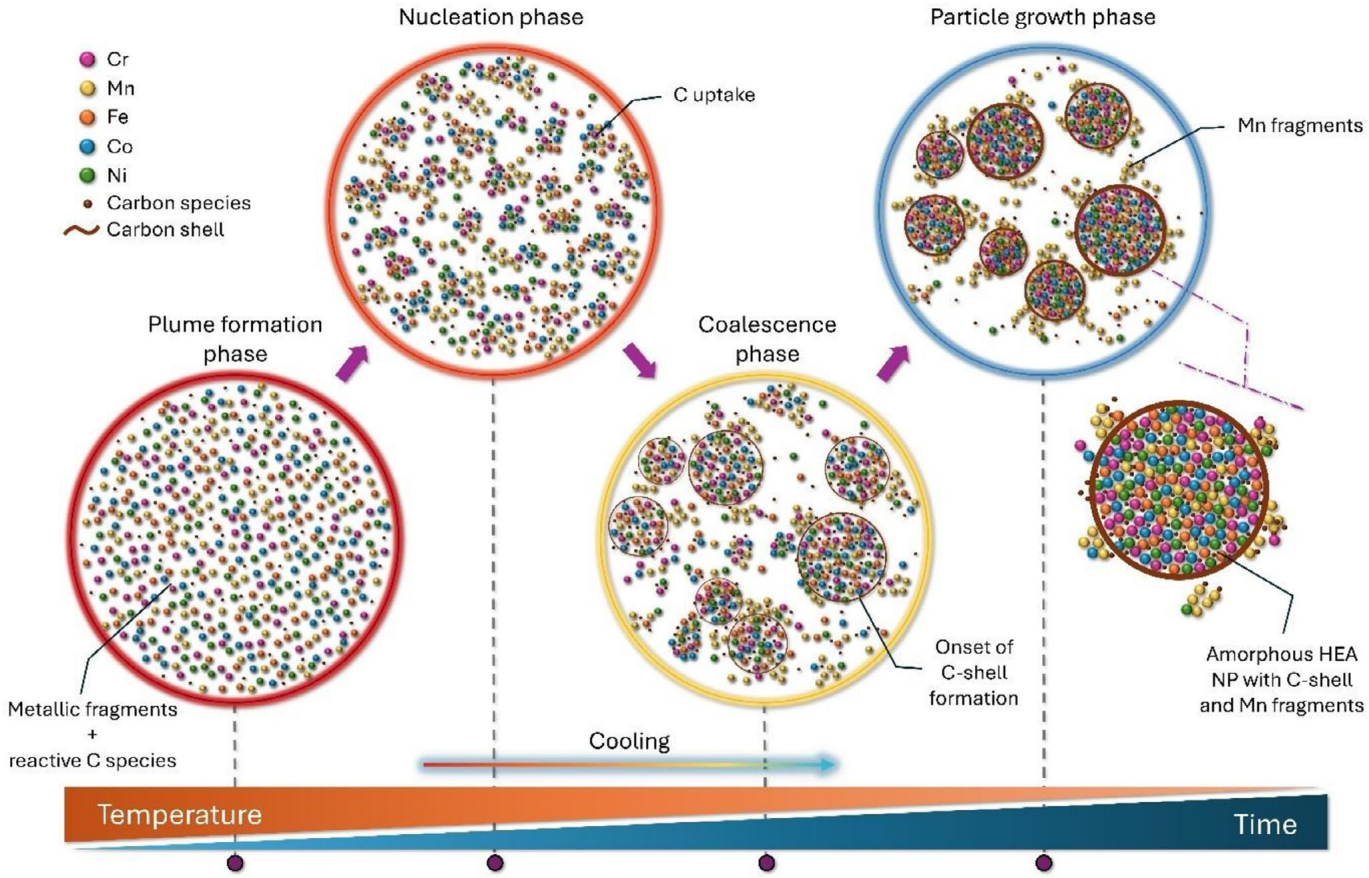
Schematic illustration of the NP formation steps during laser ablation of CrMnFeCoNi target in acetonitrile, leading to amorphous, carbon‐doped HEA nanoparticles with graphitic carbon shells surrounded by Mn‐rich fragments.

Collectively, the compositional analyses by EDS, XPS, and APT agree and provide a thorough basis to rationalize the formation of amorphous HEA nanoparticles doped with carbon and with graphitic carbon shells surrounded by Mn‐rich fragments in acetonitrile. **Figure** **5** sketches our proposed mechanism. The laser pulse focused on the alloy target creates a plume of the target's elements.^[^ ^29^ ^]^ In multi‐elemental targets, the distribution of each element within the plume is dictated by its physical properties, even when the target is a solid solution. For ultrashort‐pulsed laser ablation in liquid, atomistic simulations predicted that rapid deceleration of the ablation plume by the liquid environment results in the formation of a transient hot and dense metal region at the front of the plume. This hampers the mixing of elements and contributes to the stratification of the plume in the emerging cavitation bubble.^[^ ^60^ ^]^ The pressure at the shockwave front during nanosecond‐pulsed laser ablation in organic solvents might also affect the intensity of plume‐solvent vapor mixing.^[^ ^61^ ^]^ Experimental identification of the main elemental mixing processes during laser ablation proved the benefits of longer laser pulses for mixing Fe and Co.^[^ ^62^ ^]^ This motivated our use of nanosecond pulses herein, and it provided good and size‐independent elemental mixing, except for Mn.

The slight loss of Mn during NP consolidation may be linked to thermophysical phenomena that are more prominent at longer pulse durations, which supply energy to the plume up to six orders of magnitude longer than in femto‐ or picosecond laser ablation.^[^ ^63^ ^]^ Out of the five elements, Mn has the lowest latent heat of vaporization, the lowest melting point, and the highest vapor pressure, potentially leading to preferential separation within the ablation plume.

Concurrently, plume‐solvent interactions trigger the decomposition of organic molecules, generating active carbon species along with gases and byproducts.^[^ ^44^ ^]^ This process creates an opportunity for interaction between ablated metallic fragments within the plume and the reactive carbon species, facilitating carbon uptake (**Figure** **5**). The heat of the plume is transferred to the surrounding liquid, creating a vapor layer that encapsulates the ablated metallic content, initiating the formation of a cavitation bubble. Alloy NPs in the plume experience initial thermal quench rates of 10^13^ K s^−1^, remaining above 10^12^ K s^−1^ until undercooling occurs at 1–2 ns, as observed in FeNi alloy ablation in water.^[^ ^64^ ^]^ Larger NPs with slower cooling rates may require tens of ns to reach homogeneous crystallization thresholds, ultimately resulting in rapid solidification triggered by deep undercooling.^[^ ^64^ ^]^

The presence of reactive metals like Cr, Fe, and Mn in the ablation plume, each with relatively high carbon solubility at their melting temperatures, respectively ≈33, ≈12, and ≈7 at.%,^[^ ^45^, ^65^ ^]^ promotes the retention of carbon species within the multimetallic plume. As cooling progresses, nucleation of the metallic species happens through the attachment of neighboring atoms. Two competing processes then occur simultaneously during condensation: i) the coalescence of metallic fragments onto the growing nuclei within the plume,^[^ ^64^ ^]^ and ii) the outward movement of excess carbon dissolved in the NPs toward their surface, initiating the formation of the carbon shell that can hinder further growth.^[^ ^44^, ^49^ ^]^ The observed rugged morphology of the formed NPs with outer carbon shells indicates the simultaneous occurrence of these competing processes. Mn, with its heterogeneous distribution within the ablation plume, would be the last element to integrate on the forming nanoparticle surface. Under rapid cooling, the excess dissolved carbon migrates to the NP's surface, initiating the formation of the carbon shell. As the carbon shell begins to form, we could imagine that it competes with Mn coalescence, causing Mn to adhere to the developing carbon layer. With the subsequent collapse of the cavitation bubble, an onion‐like graphitic carbon layer grows, supplemented by available carbon in the surrounding liquid and Mn‐rich fragments attached^[^ ^30^, ^44^ ^]^ (Figure 5).

The plasma plume created by laser irradiation on the target surface exhibits extreme conditions, with initial temperatures in the order of 10^3^ K, pressures of 10^10^ Pa, and densities of 10^22^ atoms cm^−3^,^[^ ^66^ ^]^ cooling within a nanosecond time scale.^[^ ^64^ ^]^ Under such dynamic conditions, achieving thermodynamic equilibrium solidification becomes challenging, and the supersaturation of carbon into the metallic system could further impede the crystallization processes. Further studies supporting the formation of amorphous structures and enhanced glass‐forming ability in alloys with the presence of carbon can be found.^[^ ^36^, ^67^, ^68^ ^]^ A similar mechanism is observed in the case of HEA NPs in acetonitrile, where the rapid cooling conditions during ablation kinetically hinder the outward diffusion of carbon, thereby stabilizing an amorphous structure. Additionally, the incorporation of nitrogen, which is a known byproduct during ablation in acetonitrile,^[^ ^49^ ^]^ into the metallic constituents could further strengthen the formation of amorphous structures. The nitrogen concentration was found to be <0.2 at% from the APT analysis, and STEM‐EDS of the particle volume showed no significant contribution (Figure S2, Supporting Information). However, the XPS spectrum of HEA NPs synthesized in acetonitrile exhibits a peak in the N1s region (Figure S14, Supporting Information). Studies have reported on the N‐doping of the surface carbon layers during laser ablation synthesis in acetonitrile,^[^ ^49^, ^69^, ^70^ ^]^ which appears also to be observed in this case.

### 2.4 Tuning Carbon Doping and Carbon Shell Formation by Organic Solvent Selection

We extended our investigation to colloidal HEA NPs synthesized in acetone and ethanol to examine if these mechanisms can be extended to other organic solvents (see Supporting Information). Smaller NPs have rugged morphology and transition to regular spherical shapes in larger acetone‐synthesized particles, while spherical particles are widely observed in ethanol (Figures S9 and S12, Supporting Information). While HEA NPs synthesized in acetone maintain an amorphous structure, those in ethanol exhibit partial crystallization of the fcc phase (Figures S9 and S12, Supporting Information). The carbon shell thickness decreases from acetonitrile to acetone to ethanol (Figure S13, Supporting Information). XPS and STEM‐EDS composition analyses reveal reduced Mn loss in particle volumes and near‐stoichiometric distribution of other principal elements in both acetone and ethanol samples (Figures S11 and S14, Supporting Information). From these observations, the particle formation mechanism rationalized for acetonitrile can be extended to acetone and ethanol in terms of carbon supersaturation and the respective dynamics of carbon shell formation and metallic coalescence during particle formation.

**Figure 6:**
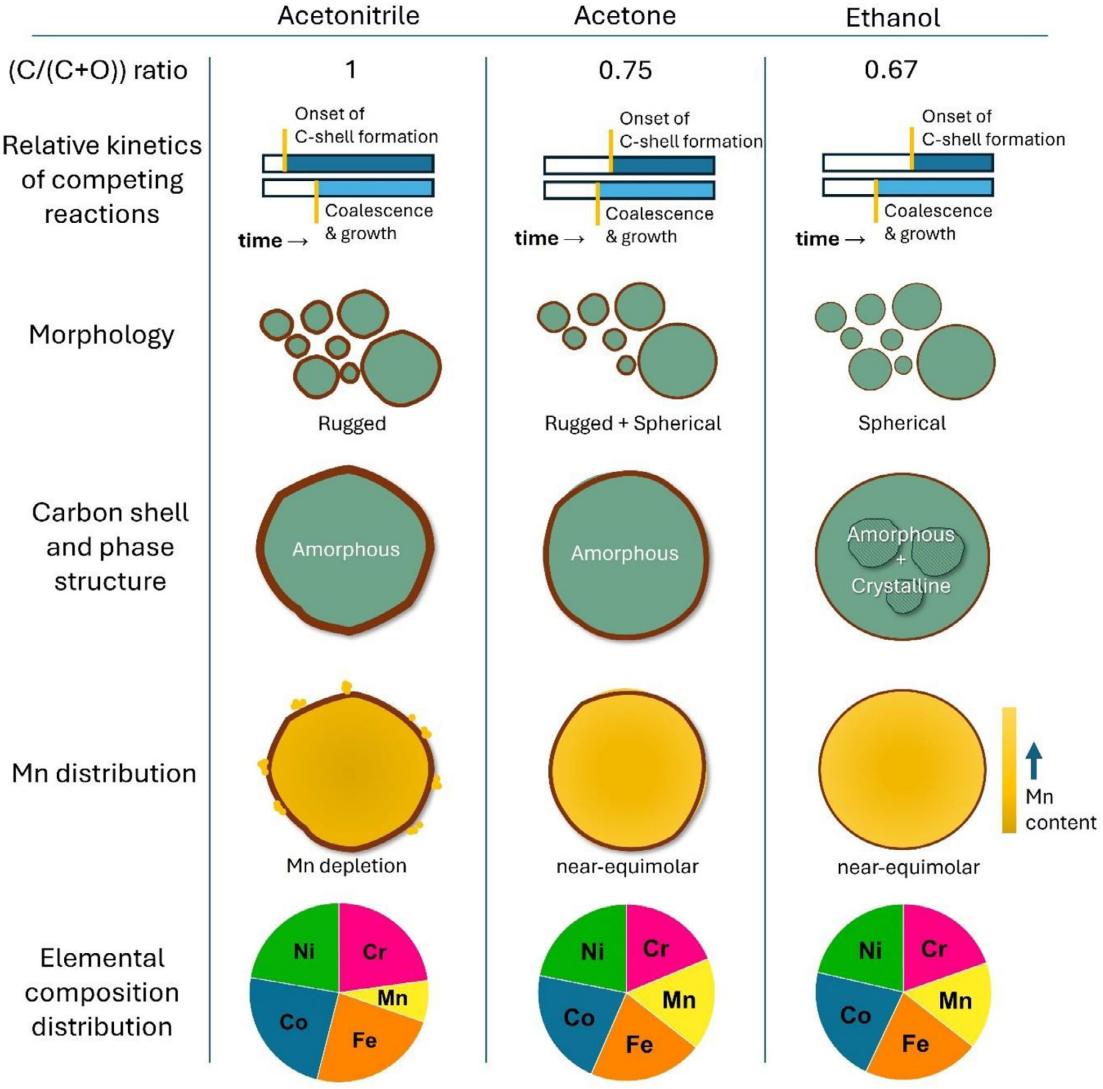
Schematic illustration comparing the mechanistic changes, particle morphology, structure, and compositional distribution variations of HEA NPs synthesized in acetonitrile, acetone, and ethanol.

**Figure** **6** schematically compares the mechanistic changes, particle morphology, structure, and compositional distribution variations of HEA NPs synthesized in acetonitrile, acetone, and ethanol. A previous report on LPSC of Fe nanoparticles^[^ ^71^ ^]^ had indicated an influence of the carbon fraction (C/(C+O)) in the solvent, which varies for ethanol (0.67), acetone (0.75), and acetonitrile (1.0) on the crystallinity of the formed NPs. Similarly, we observe less carbon incorporation during ablation in acetone and ethanol, indicated by thin carbon shell formation and insufficient carbon supersaturation to kinetically hinder crystallization compared to acetonitrile.

The carbon supersaturation in the ablation plume depends on the C/(C+O) ratio of the solvent and is hence a critical factor that mediates the competing processes of carbon shell formation and metallic constituent coalescence during nanoparticle condensation (Figure 6). In acetonitrile, where carbon supersaturation is highest, amorphous NPs with thicker carbon shells and rugged morphology are formed. The carbon shell formation dominates over the coalescence process, resulting in reduced Mn content, with the lost Mn found attached as fragments outside the shell (Figure 5). Acetone, with lesser carbon supersaturation, forms amorphous NPs with carbon shells of reduced thickness and produces a mixed morphology of larger spherical particles and smaller rugged ones. In this case, the metallic coalescence moderately precedes the carbon shell formation, preventing Mn loss. A similar effect is observed with ethanol, where partial crystallization occurs with thin carbon shells around the particles. Coalescence of metallic constituents dominates over carbon shell formation where Mn depletion is minimal, with other elements distributed close to the expected stoichiometric values. The ultrafast cooling of the ablation plume in the high fluence regime of pulsed laser synthesis quickly freezes the molten NP droplets and can explain why the composition is mostly independent of the particle size.^[^ ^64^ ^]^

Solvent‐dependent carbon doping via reactive laser ablation presents multiple tuning opportunities for synthesizing functional high‐entropy nanoalloys. Despite HEAs often exhibiting size‐dependent compositional variations and post‐synthesis phase separations, pulsed‐laser ablation enables the robust compositional design of nanoparticles independent of their size.^[^ ^72^, ^73^, ^74^ ^]^ Amorphous phase formation as a result of carbon doping in such nanoalloys promotes their use in thermal catalysis up to temperatures of 350 °C with demonstrated enhanced catalytic activity and stability due to abundant unsaturated atomic coordination and fast electron transfer.^[^ ^36^, ^75^ ^]^ Additionally, hindered crystallization during particle formation prevents preferential phase separation or short‐range ordering, thereby preserving the homogeneous chemical environment characteristic of multicomponent alloys. The carbon shell formation potentially improves conductivity during electrocatalysis while enhancing stability through a confinement effect that prevents elemental dissolution under reaction conditions.^[^ ^76^ ^]^ Furthermore, the ability to modulate the Mn content on the particle's surfaces through solvent selection serves as a valuable tool in catalyst design, as surface Mn concentration has been identified as an activity descriptor for specific electrocatalytic reactions.^[^ ^21^, ^77^ ^]^ Finally, the kinetically driven particle formation occurring during laser ablation presents a notable advantage over other synthesis methods, as evidenced by the absence of preferential phase separation or island formation within the HEA NPs despite the substantial loss of one element. Based on insights gathered from this study, future research may potentially leverage the distinct pyrolysis behavior of different solvents, as they exhibit distinct gas formation rates and produce varying decomposition products.^[^ ^43^, ^44^, ^78^, ^79^ ^]^ The nature and constitution of these decomposition byproducts could significantly influence the formation mechanisms and nanoparticles’ characteristics,^[^ ^71^, ^80^, ^81^ ^]^ making this an important area for future investigation.

### 2.5 Elemental Dissolution Studies

The amorphous structure of the HEA nanoparticles and the prominent carbon shell surrounding them could potentially influence catalytic behavior, particularly in electrocatalysis. To gain further insights, we performed preliminary scanning flow cell measurements with inductively coupled plasma–mass spectrometry (SFC–ICP–MS)^[^ ^82^, ^83^, ^84^ ^]^ and investigated metal dissolution under applied potential. A catalyst ink was prepared from dried nanoparticles and drop‐cast onto a glassy carbon electrode for SFC–ICP–MS measurements in 0.01 m H_2_SO_4_ solution to introduce harsh dissolution conditions (see methods).

**Figure 7:**
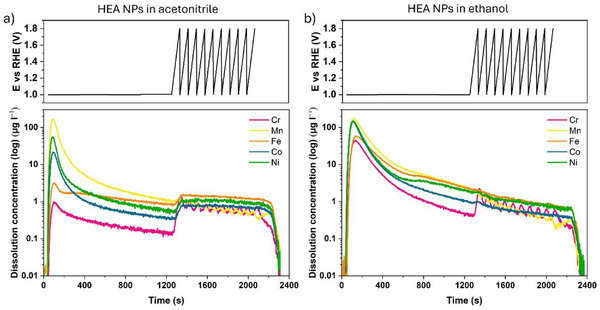
Element‐specific dissolution studies of HEA NPs synthesized in acetonitrile and ethanol using SFC–ICP–MS. a,b) Applied potential and the corresponding online ICP‐MS dissolution profiles of dissolved metal ions as a function of time of the NPs in acetonitrile and ethanol, respectively.

**Figure** **7** presents SFC–ICP–MS results comparing HEA NPs synthesized in acetonitrile and ethanol. Significant elemental dissolution was observed upon contact with the electrolyte under an applied potential of 1 V vs. reversible hydrogen electrode (RHE), which then minimized during the 1200 s hold time and OER cycling. Mn dissolution was highest in both samples during electrolyte contact. Notably, dissolution amounts of other elements differed drastically between acetonitrile and ethanol samples, though with similar trends. Following Mn, Ni exhibited the second‐highest dissolution, followed by Co, Fe, and Cr.

For HEA NPs synthesized in acetonitrile, Mn dissolution amounts to ≈67.5% and Ni dissolution ≈22.3% while Co, Fe, and Cr amount to ≈8.5%, 1.3% and 0.4%, respectively (Figure 7a). However, in the ethanol sample, Mn, Ni, and Co dissolution reach 30.9%, 25.8% and 25.6%, respectively, while Fe and Cr amount to 9.9% and 7.8% (Figure 7b). The highest Mn dissolution can be attributed to Mn‐rich fragments found outside the carbon shell in the acetonitrile sample (Figures S9–S13, Supporting Information) and the Mn‐rich surface layer in the ethanol sample (Figure S20, Supporting Information). The differences in the dissolution of other elements can be attributed to variations in the carbon shell thickness. The thick carbon shells surrounding HEA NPs synthesized in acetonitrile (Figure 1; Figure S19, Supporting Information) can potentially prevent dissolution of constituent elements, while very thin carbon shells in ethanol samples (Figures S18 and S19, Supporting Information) cannot adequately provide such protection. Additionally, the effect of the highly amorphous structure of the acetonitrile sample compared to the partially crystalline ethanol sample could influence dissolution behavior, which warrants further investigation in future studies.

In addition, after maintaining a constant potential of 1 V vs. RHE for 1200 s, potential sweeps representing OER cycling from 1 V to 1.8 V vs. RHE were performed. Elemental dissolution remained low during this stage, with Fe being the highest, followed by Ni, Cr, and Mn. Co‐dissolution was relatively higher than Cr in the acetonitrile sample, while remaining lowest in the ethanol sample (Figure 7a,b). An interesting dissolution pattern of Cr and Mn as a function of applied potential was observed in both samples. Cr dissolution began when the potential was increased to 1.8 V, followed by Mn with a slight delay. Cr and Mn dissolution patterns alternated during OER sweeps in both samples, while Fe, Ni, and Co dissolution remained nearly constant (Figure 7a,b). The alternating trend of Cr and Mn dissolution observed can be attributed to transient dissolution from the enriched subsurface to the surface layer during OER cycling, reflecting transitions between activated and deactivated states of the electrocatalyst surface under applied potential.^[^ ^77^ ^]^ Upon continued cycling, the active surface layer might undergo exfoliation, followed by dissolution of Cr and Mn from the subsurface layer, which is governed by several factors such as the potential range, electrolyte concentration, solution pH, etc.^[^ ^77^, ^85^, ^86^, ^87^ ^]^ In the ethanol sample, the contact dissolution peak tail contribution overshadowed Fe and Ni dissolution curves, depicting a decreasing trend (Figure 7b).

Overall, preliminary SFC–ICP–MS measurements of acetonitrile and ethanol samples highlight the effects of carbon shell thickness differences and surface composition variations on dissolution behavior under reaction conditions. This motivates further detailed studies on the electrochemical behavior of these HEA nanoparticles synthesized in different solvent media to potentially tailor their performance and stability.

## 3 Conclusion

The synthesis of amorphous nanoparticles based on the Cantor alloy using pulsed laser ablation in selected organic solvents is demonstrated. The kinetic‐control nature of the process provides high compositional robustness, independent of the particle size, as well as thermally quite stable particles. The interaction of reactive carbon species with the metallic plume at the nanoseconds‐prolonged ultrahigh temperatures and the HEA's supersaturated carbon content causes the stabilization of the amorphous structures, which is evidenced by in situ TEM heating experiments. The solvent molecule determines the carbon doping, and part of the supersaturated excess carbon migrates to the surface during condensation to form the onion‐like graphitic carbon shells. The degree of solvent‐dependent carbon supersaturation alters the time scales of the competing reactions—carbon shell formation and metallic coalescence—thereby altering composition distribution and carbon shell thickness, which is mechanistically rationalized through a proposed particle formation mechanism. Additionally, reduced carbon availability for reaction with the ablation plume results in spherical morphology and partial crystallization in ethanol. The robustness of the catalyst under reaction conditions (OER) benefits from the thicker carbon shells, as evidenced in element‐specific dissolution profiles. Hence, during nanosecond‐pulsed laser synthesis of these carbon‐affine HEA colloids, solvent molecule selection allows for dictating the degree of amorphization (by carbon doping) and the carbon shell thickness. This provides a novel pathway for tuning nanoparticle morphology, structure, and surface composition via reactive LSPC.

## 4 Experimental Section

### Target Preparation

The bulk equimolar CrMnFeCoNi HEA alloy was produced from pure metals by melting and casting using a vacuum induction furnace. Blocks of dimension 10 × 20 × 60 mm^3^ were machined from the cast block, which were then hot‐rolled at 950 °C to reduce the thickness from 10 to 5 mm (50% reduction ratio). After the hot rolling process, the sheets were homogenized at 1200 °C for 3 h in an Argon atmosphere, followed by water‐quenching. The sheets were then subjected to a cold rolling process to further reduce the thickness from 5 to 2.5 mm (50% reduction ratio). A final annealing step was carried out at 900 °C for 1 h in an Argon atmosphere, followed by water‐quenching.

### Pulsed‐Laser Ablation in Liquids

The nanoparticle synthesis was performed using pulsed‐laser ablation with a nanosecond EdgeWave GmbH laser with a wavelength of 1064 nm, pulse duration of 8 ns, repetition rate of 5 kHz, and pulse energy of 35 mJ. A polished bulk HEA target was positioned in a flow chamber, where distilled and nitrogen‐bubbled organic solvents (acetonitrile, ethanol, or acetone) flowed continuously at ≈50 mL min^−1^. Colloidal nanoparticles were produced by scanning the focused laser beam across the bulk target in a rectangular pattern.

### Materials Characterization

Qualitative phase analysis of the bulk target and nanoparticles was performed using X‐ray diffraction (XRD) measurements (Rikaku Smartlab 9 kW diffractometer) with Cu Kα radiation (λ = 0.15406 nm), where the 2θ angle was varied between 20° and 120° with a step size of 0.01° and a scan speed of 1° min^−1^. A voltage of 45 kV and a current of 200 mA were used during the measurements.

Composition analysis of the bulk target was performed using scanning electron microscopy (SEM) coupled with energy dispersive X‐ray spectroscopy (EDS) (Zeiss Sigma). An accelerating voltage of 15 kV and a probe current of 7.7 nA were used.

Raman spectra of the dried HEA NP powders were recorded using a confocal Raman microscope from Oxford Instruments/WITec (WITec alpha300M+, Ulm, Germany) and the integrated 532 nm laser (approx. power of 9mW), a UHTS 300 spectrograph (600 grooves per mm grating), and a CCD detector (DU401A‐BV‐352, Andor, UK). The Raman spectra were recorded at 3 different spots with optimized exposure times and 10 accumulations.

The oxidation states and relative surface elemental composition analysis of the nanoparticles were performed by X‐ray photoelectron spectroscopy (XPS) using a VersaProbe IITM from Ulvac‐Phi using the Al‐Kα line at 1486.6 eV and a spot size of 100 µm at an energetic resolution of 0.5 eV. A hemispherical analyzer (with an angle of 45° between the sample surface and the analyzer) and a dual‐beam charge neutralization were used, whereby the detector was operated at a pass energy of 23 eV. By centrifugation, the as‐synthesized amorphous HEA NPs of different sizes were separated into five classes (d>100 nm, 100<d<50 nm, 50<d<20 nm, 20<d<10 nm and d<10 nm), and drop cast onto a quartz substrate for XPS measurements. CasaXPS software was used to perform the peak deconvolutions with a Shirley^[^ ^88^ ^]^ background applied to the individual peak fits. The acquired high‐resolution XPS spectra were calibrated according to the binding energy of adventitious carbon (284.8 eV) determined during deconvolution of the C 1s spectrum of each sample^[^ ^89^ ^]^. The constraints used during the peak fitting of the 2p and 3p spectra of all elements and the respective peak fits of all samples can be found in the Supporting Information. Auger contributions from Fe, Co, and Ni were considered during the 2p peak deconvolutions. Both 2p^3/2^ and 2p^1/2^ signals of Fe were fitted considering the Auger contribution from Co and Ni in its binding energy range.^[^ ^90^ ^]^ The peak types were chosen generally as asymmetric Lorentzian for the elementary peaks and symmetric Gauss‐Lorentzian for the oxide/hydroxide peaks. The peak areas calculated from the deconvolutions of the 2p^3/2^ signals were used for the surface composition quantification. Additionally, complementary composition quantification using the peak areas calculated from the 3p signals of the individual elements was performed. The kinetic energy and inelastic mean free path of the photoelectrons emitted by different orbitals differ,^[^ ^91^, ^92^ ^]^ and the compositional information from 3p orbitals originates from relatively deeper below the surface compared to signals from 2p orbitals. Electrons from 2p orbitals have higher binding energies, resulting in lower kinetic energies after photoemission and consequently shorter inelastic mean free paths. Conversely, 3p electrons, with their lower binding energies, possess higher kinetic energies and longer inelastic mean free paths. Hence, the signals in the 3p region contain information from deeper below the surface compared to the 2p signals. Using Fe as an example, the depth probed by 2p electron signals is ≈4–4.5 nm, while for 3p signals it is ≈6–7 nm without accounting for elastic scattering.^[^ ^50^, ^51^, ^52^ ^]^ Similar values can be assumed for other elements.

Structure and morphology analyses of the nanoparticles were performed using transmission electron microscopy (TEM) bright field (BF) imaging (Thermo Fischer Titan Themis G2) operated at 300 kV coupled with selected area electron diffraction (SAED). The TEM specimens were prepared by drop casting 5 µL of colloidal nanoparticles on a lacey‐carbon grid, followed by drying in air. The TEM grids were plasma‐cleaned for 20 s before analysis. Composition analyses of the nanoparticles were performed using scanning transmission electron microscopy (STEM) combined with energy dispersive X‐ray spectroscopy (EDS) (Thermo Fischer Titan Themis 300 – probe corrected). The microscope was operated in STEM mode at an accelerating voltage of 300 kV, camera length of 100 mm, beam convergence angle of 23.8 mrad, and for imaging, a bright field detector and a high‐angle annular dark field (HAADF) detector were used. The Velox 3.6.0 software from Thermo Fischer Scientific was used for image and EDS acquisition. The Kα peaks of the elements Cr, Mn, Fe, Co, and Ni were used for elemental mapping and respective quantifications. A standard Cliff‐Lorimer (K‐factor) quantification, including absorption correction, was used for EDS quantification. Each EDS map was an accumulation of at least 300 frames of image size 1024 × 1024 pixels with dwell times varying between 10 to 20 µs to ensure sufficient intensity counts on the spectra for quantification.

### In Situ Heating Studies

In situ heating studies of the CrMnFeCoNiC_x_ NPs synthesized in acetonitrile were performed using a Thermo Fischer Titan Themis G2 TEM instrument operated at 300 kV. An in situ chip holder with amorphous SiN_x_ windows (Nano‐Chips Wildfire Double Tilt – DENSsolutions) was used for the analysis, where the colloidal NPs were drop‐cast and air dried. The in situ chip was plasma‐cleaned for 20 s before analysis. The sample was heated to 600 °C in steps of 100 °C until 300 °C with a heating rate of 1 °C s^−1^ and in steps of 50 °C until 600 °C with a heating rate of 0.2 °C s^−1^. At each temperature step, a holding time of 60 s was used to stabilize the sample before imaging was performed.

### Atom Probe Tomography

A dual beam scanning‐electron microscope – Ga‐ion focused ion beam (SEM‐FIB) microscope (Thermo Fischer Helios 600i) was used to prepare the needle‐shaped atom probe tomography (APT) specimens from the bulk HEA target by the in situ lift‐out method,^[^ ^93^ ^]^ followed by annular milling until the tip radius is less than 100 nm. The nanoparticle aggregates were embedded in a Ni matrix using co‐electrodeposition on a Cu substrate to aid the preparation of APT specimens. Detailed specimen preparation steps can be found here.^[^ ^94^ ^]^ The prepared needle‐shaped APT specimens from the nanoparticle aggregate regions were in situ‐coated with metallic Cr for enhanced yield.^[^ ^56^, ^95^ ^]^ APT measurements were carried out using a Cameca LEAP 5000 XR, with a base temperature of 60 K, pulsed UV‐laser mode at an energy of 60 pJ per pulse, a target detection rate of 0.5%, and a repetition rate of 125 kHz. Data reconstruction and post‐processing were done using the software AP Suite by Cameca Instruments following the voltage‐based reconstruction protocol.

### Scanning Flow Cell Measurements

The electrochemical experiments were performed in a micro‐electrochemical scanning flow cell (SFC)^[^ ^82^, ^83^ ^]^ made of polycarbonate (Makrolon) using a Gamry Reference 600 potentiostat. The counter electrode (Pt‐wire, 0.5 mm, 99,997%, Alfa Aesar) was placed in the inlet channel, and the reference electrode (Ag/AgCl/3 m KCl) in the outlet channel of the SFC. The diameter of the channels was 1.9 mm. An electrolyte solution of 0.01 m H_2_SO_4_ that was prepared from suprapure H_2_SO_4_ (96%, Merck) and ultrapure water (PureLab Flex2, Elga, 18MΩ cm^−1^, TOC < 3ppb) was used. The electrolyte was pumped with a flow rate of ≈380 µL min^−1^ through the cell and subsequently introduced into an inductively coupled plasma – mass spectrometer (ICP‐MS, NexION 300X, Perkin Elmer) for time‐resolved analysis of the concentration of dissolved ions (^52^Cr, ^55^Mn, ^56^Fe, ^59^Co, ^60^Ni).

The catalyst ink was prepared by mixing 4 mg catalyst powder with 4 mL of a solution mixture containing isopropanol (1 mL), MilliQ water (3 mL), and Nafion (30 µL). 1.4 µL of the ink was drop‐casted onto a polished glassy carbon plate, which acts as the working electrode, enclosed by the opening of the SFC. The detected intensities of the metal ions were then analyzed with respect to an internal standard for compensation of physical interferences. For this purpose, ^74^Ge in 0.01 m H_2_SO_4_ with a concentration of 50 µg L^−1^ was used for all elements. This solution was added to the electrolyte via a Y‐connector behind the SFC.

A four‐point calibration of the ICP‐MS was performed every day before the measurements and used to convert the detected intensities to the concentration of the dissolved ions in the electrolyte. The delay time of ≈45 s (varies between 43 and 52 s) between dissolution of ions at the working electrode and the detection at the ICP‐MS was compensated by synchronizing the time scale.

The electrochemical protocol started with a potentiostatic hold at 1 V vs. the reversible hydrogen electrode (RHE). During this hold, the sample was contacted under potential control. After the first contact dissolution peak had subsided (1200 s), 10 ramps from 1 to 1.8 V vs. RHE with a scan rate of 10 mV s^−1^ were performed.

## Conflict of Interest

The authors declare no conflict of interest.

## Supporting information



Supporting Information

## Data Availability

The data that support the findings of this study are available from the corresponding author upon reasonable request.

## References

[1] D. Akinwande , N. Petrone , J. Hone , Nat. Commun. 2014, 5, 5678. 25517105 10.1038/ncomms6678

[2] C. Gong , K. Hu , X. Wang , P. Wangyang , C. Yan , J. Chu , M. Liao , L. Dai , T. Zhai , C. Wang , L. Li , J. Xiong , Adv. Funct. Mater. 2018, 28, 1706559.

[3] Y. Nakaya , S. Furukawa , Chem. Rev. 2023, 123, 5859. 36170063 10.1021/acs.chemrev.2c00356

[4] S. Furukawa , T. Komatsu , K. Shimizu , J. Mater. Chem. A 2020, 8, 15620.

[5] S. Schauermann , N. Nilius , S. Shaikhutdinov , H.‐J. Freund , Acc. Chem. Res. 2013, 46, 1673. 23252628 10.1021/ar300225s

[6] A. Chen , S. Chatterjee , Chem. Soc. Rev. 2013, 42, 5425. 23508125 10.1039/c3cs35518g

[7] Q. Zhang , E. Uchaker , S. L. Candelaria , G. Cao , Chem. Soc. Rev. 2013, 42, 3127. 23455759 10.1039/c3cs00009e

[8] K. Loza , M. Heggen , M. Epple , Adv. Funct. Mater. 2020, 30, 1909260.

[9] M. B. Cortie , A. M. McDonagh , Chem. Rev. 2011, 111, 3713. 21235212 10.1021/cr1002529

[10] A. K. Singh , Q. Xu , ChemCatChem 2013, 5, 652.

[11] H. Li , J. Lai , Z. Li , L. Wang , Adv. Funct. Mater. 2021, 31, 2106715.

[12] T. Löffler , A. Ludwig , J. Rossmeisl , W. Schuhmann , Angew. Chem., Int. Ed. 2021, 60, 26894. 10.1002/anie.202109212 PMC9292432 34436810

[13] K. Wang , J. Huang , H. Chen , Y. Wang , W. Yan , X. Yuan , S. Song , J. Zhang , X. Sun , Electrochem. Energy Rev. 2022, 5, 17.

[14] E. P. George , D. Raabe , R. O. Ritchie , Nat. Rev. Mater. 2019, 4, 515.

[15] Y. Xin , S. Li , Y. Qian , W. Zhu , H. Yuan , P. Jiang , R. Guo , L. Wang , ACS Catal. 2020, 10, 11280.

[16] S. Krouna , A. Acheche , G. Wang , N. O. Pena , R. Gatti , C. Ricolleau , H. Amara , J. Nelayah , D. Alloyeau , Adv. Mater. 2025, 37, 2414510. 39573892 10.1002/adma.202414510 PMC11775865

[17] L. Han , S. Zhu , Z. Rao , C. Scheu , D. Ponge , A. Ludwig , H. Zhang , O. Gutfleisch , H. Hahn , Z. Li , D. Raabe , Nat. Rev. Mater. 2024, 9, 846.

[18] B. Cantor , I. T. H. Chang , P. Knight , A. J. B. Vincent , Mater. Sci. Eng., A 2004, 375–377, 213.

[19] D. B. Miracle , O. N. Senkov , Acta Mater. 2017, 122, 448.

[20] Y. Zhang , D. Wang , S. Wang , Small 2022, 18, 2104339.

[21] T. Löffler , F. Waag , B. Gökce , A. Ludwig , S. Barcikowski , W. Schuhmann , ACS Catal. 2021, 11, 1014.

[22] Y. Chida , T. Tomimori , T. Ebata , N. Taguchi , T. Ioroi , K. Hayashi , N. Todoroki , T. Wadayama , Nat. Commun. 2023, 14, 4492. 37495632 10.1038/s41467-023-40246-5 PMC10372069

[23] Y. Sun , S. Dai , Nature Synthesis 2024, 3, 1457.

[24] L. Yang , R. He , J. Chai , X. Qi , Q. Xue , X. Bi , J. Yu , Z. Sun , L. Xia , K. Wang , N. Kapuria , J. Li , A. Ostovari Moghaddam , A. Cabot , Adv. Mater. 2025, 37, 2412337. 10.1002/adma.202412337 39473325

[25] P. Bernard , P. Stelmachowski , P. Broś , W. Makowski , A. Kotarba , J. Chem. Educ. 2021, 98, 935. 33814599 10.1021/acs.jchemed.0c01101 PMC8016114

[26] P. Mäki‐Arvela , D. Yu Murzin , Appl. Catal., A 2013, 451, 251.

[27] H. Luan , L. Huang , J. Kang , B. Luo , X. Yang , J. Li , Z. Han , J. Si , Y. Shao , J. Lu , K.‐F. Yao , Acta Mater. 2023, 248, 118775.

[28] F. Otto , A. Dlouhý , K. G. Pradeep , M. Kuběnová , D. Raabe , G. Eggeler , E. P. George , Acta Mater. 2016, 112, 40.

[29] D. Zhang , B. Gökce , S. Barcikowski , Chem. Rev. 2017, 117, 3990. 28191931 10.1021/acs.chemrev.6b00468

[30] S. Reichenberger , G. Marzun , M. Muhler , S. Barcikowski , ChemCatChem 2019, 11, 4489.

[31] R. C. Forsythe , C. P. Cox , M. K. Wilsey , A. M. Müller , Chem. Rev. 2021, 121, 7568. 34077177 10.1021/acs.chemrev.0c01069

[32] A. A. Manshina , I. I. Tumkin , E. M. Khairullina , M. Mizoshiri , A. Ostendorf , S. A. Kulinich , S. Makarov , A. A. Kuchmizhak , E. L. Gurevich , Adv. Funct. Mater. 2024, 34, 2405457.

[33] F. Waag , R. Streubel , B. Gökce , S. Barcikowski , Appl. Nanosci. 2021, 11, 1303.

[34] V. Amendola , D. Amans , Y. Ishikawa , N. Koshizaki , S. Scirè , G. Compagnini , S. Reichenberger , S. Barcikowski , Chemistry – A European Journal 2020, 26, 9206. 32311172 10.1002/chem.202000686 PMC7497020

[35] A. Guadagnini , S. Agnoli , D. Badocco , P. Pastore , R. Pilot , R. Ravelle‐Chapuis , M. B. F. van Raap , V. Amendola , ChemPhysChem 2021, 22, 657. 33559943 10.1002/cphc.202100021

[36] S.‐X. Liang , L.‐C. Zhang , S. Reichenberger , S. Barcikowski , Phys. Chem. Chem. Phys. 2021, 23, 11121. 33969854 10.1039/d1cp00701g

[37] R. Rawat , N. P. Blanchard , Y. Shadangi , A. Tripathi , D. Amans , J. Phys. Chem. C 2024, 128, 19815.

[38] C. Guo , X. Hu , X. Han , Y. Gao , T. Zheng , D. Chen , X. Qiu , P. Wang , K. Xu , Y. Chen , R. Zhou , M. Zong , J. Wang , Z. Xia , J. Hao , K. Xie , J. Am. Chem. Soc. 2024, 146, 18407. 38935530 10.1021/jacs.4c03658

[39] J. Johny , Y. Li , M. Kamp , O. Prymak , S.‐X. Liang , T. Krekeler , M. Ritter , L. Kienle , C. Rehbock , S. Barcikowski , S. Reichenberger , Nano Res. 2022, 15, 4807.

[40] S. Tahir , N. Shkodich , B. Eggert , J. Lill , O. Gatsa , M. Flimelová , E. Adabifiroozjaei , N. M. Bulgakova , L. Molina‐Luna , H. Wende , M. Farle , A. V. Bulgakov , C. Doñate‐Buendía , B. Gökce , ChemNanoMat 2024, 10, 202400064.

[41] F. Waag , Y. Li , A. Rosa Ziefuß , E. Bertin , M. Kamp , V. Duppel , G. Marzun , L. Kienle , S. Barcikowski , B. Gökce , RSC Adv. 2019, 9, 18547. 35515245 10.1039/c9ra03254a PMC9064730

[42] B. Wang , C. Wang , X. Yu , Y. Cao , L. Gao , C. Wu , Y. Yao , Z. Lin , Z. Zou , Nature Synthesis 2022, 1, 138.

[43] T. Fromme , L. K. Tintrop , S. Reichenberger , T. C. Schmidt , S. Barcikowski , ChemPhysChem 2023, 24, 202300089. 10.1002/cphc.202300089 36878868

[44] T. Fromme , S. Reichenberger , K. M. Tibbetts , S. Barcikowski , Beilstein J. Nanotechnol. 2024, 15, 638. 38887526 10.3762/bjnano.15.54 PMC11181208

[45] D. Zhang , C. Zhang , J. Liu , Q. Chen , X. Zhu , C. Liang , ACS Appl. Nano Mater. 2019, 2, 28.

[46] D. Zhang , W. Choi , Y. Oshima , U. Wiedwald , S.‐H. Cho , H.‐P. Lin , Y. K. Li , Y. Ito , K. Sugioka , Nanomaterials 2018, 8, 631. 30127303 10.3390/nano8080631 PMC6116272

[47] B. Pang , Y. Ma , Z. Tian , J. Liu , S. Wu , D. Teng , P. Li , C. Liang , J. Colloid Interface Sci. 2021, 585, 452. 33268061 10.1016/j.jcis.2020.10.026

[48] N. D. Stepanov , N. Yu Yurchenko , M. A. Tikhonovsky , G. A. Salishchev , J. Alloys Compd. 2016, 687, 59.

[49] H. J. Jung , M. Y. Choi , Appl. Surf. Sci. 2018, 457, 1050.

[50] S. Tanuma , C. J. Powell , D. R. Penn , Surf. Interface Anal. 1991, 17, 911.

[51] S. Tanuma , C. J. Powell , D. R. Penn , Surf. Interface Anal. 2011, 43, 689.

[52] G. Greczynski , L. Hultman , J. Appl. Phys. 2022, 132, 011101.

[53] A. Ferrari , F. Körmann , Appl. Surf. Sci. 2020, 533, 147471.

[54] D. Chatain , P. Wynblatt , Comput. Mater. Sci. 2021, 187, 110101.

[55] P. Wynblatt , D. Chatain , Phys. Rev. Mater. 2019, 3, 054004.

[56] T. M. Schwarz , E. Woods , M. P. Singh , X. Chen , C. Jung , L. S. Aota , K. Jang , M. Krämer , S.‐H. Kim , I. McCarroll , B. Gault , Microsc. Microanal. 2024, 30, ozae006. 10.1093/mam/ozae006 38366381

[57] F. De Geuser , B. Gault , Acta Mater. 2020, 188, 406.

[58] B. Gault , B. Klaes , F. F. Morgado , C. Freysoldt , Y. Li , F. D. Geuser , L. T. Stephenson , F. Vurpillot , Microsc. Microanal. 2022, 28, 1116. 10.1017/S1431927621012952 34666868

[59] F. Vurpillot , A. Bostel , D. Blavette , Appl. Phys. Lett. 2000, 76, 3127.

[60] C.‐Y. Shih , C. Chen , C. Rehbock , A. Tymoczko , U. Wiedwald , M. Kamp , U. Schuermann , L. Kienle , S. Barcikowski , L. V. Zhigilei , J. Phys. Chem. C 2021, 125, 2132.

[61] V. Coviello , C. Reffatto , M. W. Fawaz , B. Mahler , A. Sollier , B. Lukic , A. Rack , D. Amans , V. Amendola , Adv. Sci. 2025, 12, 2416035. 10.1002/advs.202416035 PMC12140356 40285623

[62] F. Waag , W. I. M. A. Fares , Y. Li , C. Andronescu , B. Gökce , S. Barcikowski , J. Mater. Sci. 2022, 57, 3041.

[63] C.‐Y. Shih , M. V. Shugaev , C. Wu , L. V. Zhigilei , Phys. Chem. Chem. Phys. 2020, 22, 7077. 32196057 10.1039/d0cp00608d

[64] C. Chen , L. V. Zhigilei , Appl. Phys. A 2023, 129, 288.

[65] H. Lekkishef , Diagrams of the State of Double Metal Systems, Chemical Industry Press, Beijing 2009.

[66] V. Amendola , M. Meneghetti , Phys. Chem. Chem. Phys. 2013, 15, 3027. 23165724 10.1039/c2cp42895d

[67] S.‐X. Liang , S. Salamon , S. Zerebecki , L.‐C. Zhang , Z. Jia , H. Wende , S. Reichenberger , S. Barcikowski , Scr. Mater. 2021, 203, 114094.

[68] F. Kong , A. Inoue , F. Wang , C. Chang , Coatings 2024, 14, 118.

[69] H.‐R. Cheng , H. S. Kim , Energy & Environmental Materials 2025, 70103.

[70] F. Ullah , R. Karimi , H. M. Fruehwald , R. D. L. Smith , J. Sanderson , K. P. Musselman , Adv. Mater. 2025, 09060. 10.1002/adma.202509060 PMC12651130 40810615

[71] A. Kanitz , J. S. Hoppius , M. del Mar Sanz , M. Maicas , A. Ostendorf , E. L. Gurevich , ChemPhysChem. 2017, 18, 1155. 28188671 10.1002/cphc.201601252

[72] F. Calvo , Phys. Chem. Chem. Phys. 2023, 25, 18439. 37401561 10.1039/d3cp01869e

[73] P.‐C. Chen , M. Gao , C. A. McCandler , C. Song , J. Jin , Y. Yang , A. L. Maulana , K. A. Persson , P. Yang , Nat. Nanotechnol. 2024, 19, 775. 38429491 10.1038/s41565-024-01626-0

[74] S. Moniri , Y. Yang , J. Ding , Y. Yuan , J. Zhou , L. Yang , F. Zhu , Y. Liao , Y. Yao , L. Hu , P. Ercius , J. Miao , Nature 2023, 624, 564. 38123807 10.1038/s41586-023-06785-z

[75] J. Kang , X. Yang , Q. Hu , Z. Cai , L.‐M. Liu , L. Guo , Chem. Rev. 2023, 123, 8859. 37358266 10.1021/acs.chemrev.3c00229

[76] J. M. Yoo , H. Shin , D. Y. Chung , Y.‐E. Sung , Acc. Chem. Res. 2022, 55, 1278. 35436084 10.1021/acs.accounts.1c00727

[77] C. Luan , D. Escalera‐López , U. Hagemann , A. Kostka , G. Laplanche , D. Wu , S. Cherevko , T. Li , ACS Catal. 2024, 14, 12704.

[78] S. C. Moldoveanu , Pyrolysis of Organic Molecules: Applications to Health and Environmental Issues, Vol. 28, Elsevier, Amsterdam 2009.

[79] T. Fromme , R. Müller , L. Krenz , L. K. Tintrop , I. Sanjuán , T. C. Schmidt , K. M. Tibbetts , C. Andronescu , S. Reichenberger , S. Barcikowski , J. Phys. Chem. C 2025, 129, 2953.

[80] K. Suehara , R. Takai , Y. Ishikawa , N. Koshizaki , K. Omura , H. Nagata , Y. Yamauchi , ChemPhysChem 2021, 22, 675. 33496376 10.1002/cphc.202001000

[81] V. Amendola , P. Riello , M. Meneghetti , J. Phys. Chem. C 2011, 115, 5140.

[82] S. O. Klemm , A. A. Topalov , C. A. Laska , K. J. J. Mayrhofer , Electrochem. Commun. 2011, 13, 1533.

[83] A. K. Schuppert , A. A. Topalov , I. Katsounaros , S. O. Klemm , K. J. J. Mayrhofer , J. Electrochem. Soc. 2012, 159, F670.

[84] H. Zhao , Y. Yin , Y. Wu , S. Zhang , A. M. Mingers , D. Ponge , B. Gault , M. Rohwerder , D. Raabe , Nat. Commun. 2024, 15, 561. 38228660 10.1038/s41467-024-44802-5 PMC10792079

[85] L. D. Burke , E. J. M. O'Sullivan , J. Electroanal. Chem. Interfacial Electrochem. 1981, 117, 155.

[86] S. Rebouillat , M. E. G. Lyons , M. P. Brandon , R. L. Doyle , Int. J. Electrochem. Sci. 2011, 6, 5830.

[87] L. D. Burke , M. E. Lyons , O. J. Murphy , J. Electroanal. Chem. Interfacial Electrochem. 1982, 132, 247.

[88] D. A. Shirley , Phys. Rev. B 1972, 5, 4709.

[89] M. C. Biesinger , Appl. Surf. Sci. 2022, 597, 153681.

[90] C. D. Wanger , W. M. Riggs , L. E. Davis , J. F. Moulder , G. E. Muilenberg , Handbook of X‐ray Photoelectron Spectroscopy, Perkin‐Elmer Corporation, Eden Prairie, Minnesota, USA 1979.

[91] C. J. Powell , Journal of Vacuum Science & Technology A 2020, 38, 023209.

[92] A. Jablonski , C. J. Powell , J. Electron Spectrosc. Relat. Phenom. 1999, 100, 137.

[93] B. Gault , A. Chiaramonti , O. Cojocaru‐Mirédin , P. Stender , R. Dubosq , C. Freysoldt , S. K. Makineni , T. Li , M. Moody , J. M. Cairney , Nature Reviews Methods Primers 2021, 1, 51. 10.1038/s43586-021-00047-w PMC10502706 37719173

[94] S.‐H. Kim , P. W. Kang , O. O. Park , J.‐B. Seol , J.‐P. Ahn , J. Y. Lee , P.‐P. Choi , Ultramicroscopy 2018, 190, 30. 29680520 10.1016/j.ultramic.2018.04.005

[95] E. V. Woods , M. P. Singh , S.‐H. Kim , T. M. Schwarz , J. O. Douglas , A. A. El‐Zoka , F. Giulani , B. Gault , Microsc. Microanal. 2023, 29, 1992. 37856778 10.1093/micmic/ozad120

