# Supplementary material for: Kinetically Controlling Surface Atom Arrangements in Thermally Robust, Amorphous High‐Entropy Alloy Nanoparticles by Solvent Selection
Source: Adv Sci (Weinh). 2025 Oct 13;13(1):e10537. doi: 10.1002/advs.202510537 (PMC12767063; doi:10.1002/advs.202510537)
Supplement: Supplementary file 1 — Supporting Information [file ADVS-13-e10537-s001.docx]

# Supporting Information

# Kinetically Controlling Surface Atom Arrangements in Thermally Robust, Amorphous High-Entropy Alloy Nanoparticles by Solvent Selection

*Varatharaja Nallathambi, Se-Ho Kim, Andrea M. Mingers, Petra Ebbinghaus, Baptiste Gault, Sven Reichenberger, Dierk Raabe^*^, and Stephan Barcikowski^*^*

V. Nallathambi, S. Reichenberger, S. Barcikowski

Technical Chemistry I and Center for Nanointegration Duisburg-Essen (CENIDE), University of Duisburg-Essen, 45141 Essen, Germany

*E-mail: [stephan.barcikowski@uni-due.de](mailto:stephan.barcikowski@uni-due.de)

V. Nallathambi, S. Kim, A.M. Mingers, P. Ebbinghaus, B. Gault, D. Raabe

Max Planck Institute for Sustainable Materials, Max-Planck-Str.1, 40237 Düsseldorf, Germany

*E-mail: [d.raabe@mpie.de](mailto:d.raabe@mpie.de)

S. Kim

Department of Materials Science & Engineering, Korea University, Seoul, 02841, Republic of Korea

B. Gault

Department of Materials, Royal School of Mines, Imperial College London, London SW72AZ, United Kingdom

Keywords: Amorphous nanoparticles, high-entropy alloys, compositionally complex alloys, laser ablation, nanoparticle synthesis, in situ electron microscopy, atom probe tomography

### Structural and compositional characterization


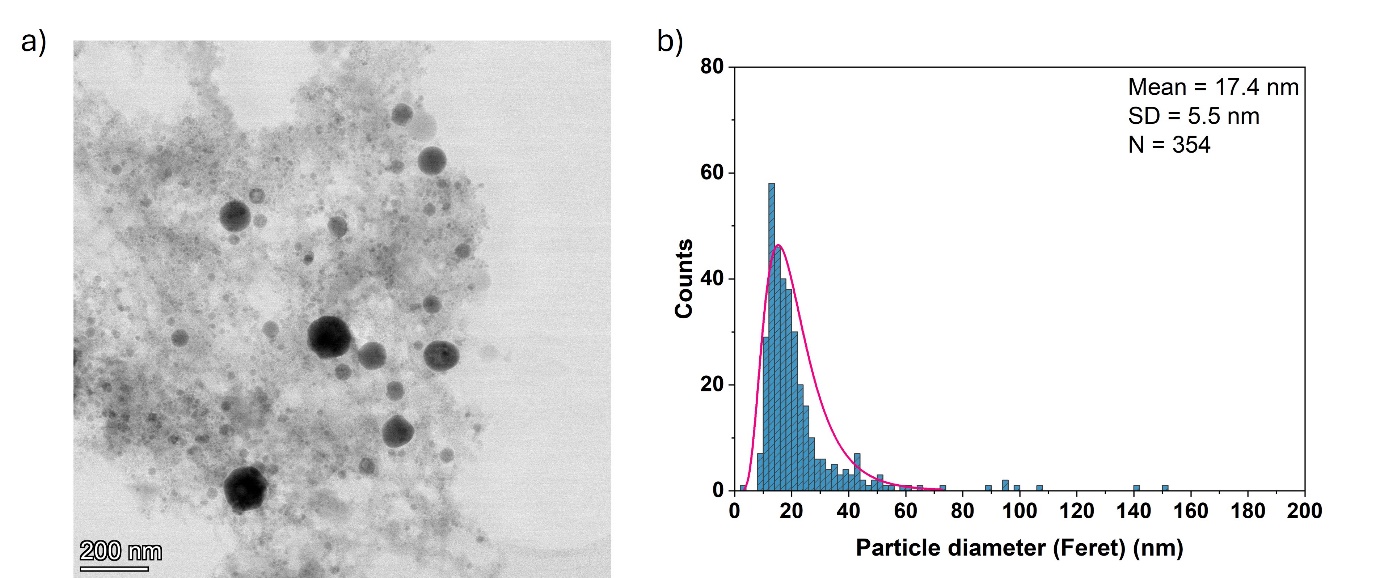


Figure S1 a) STEM bright field image of the HEA NPs synthesized in acetonitrile and b) the corresponding size distribution (lognormal fit).


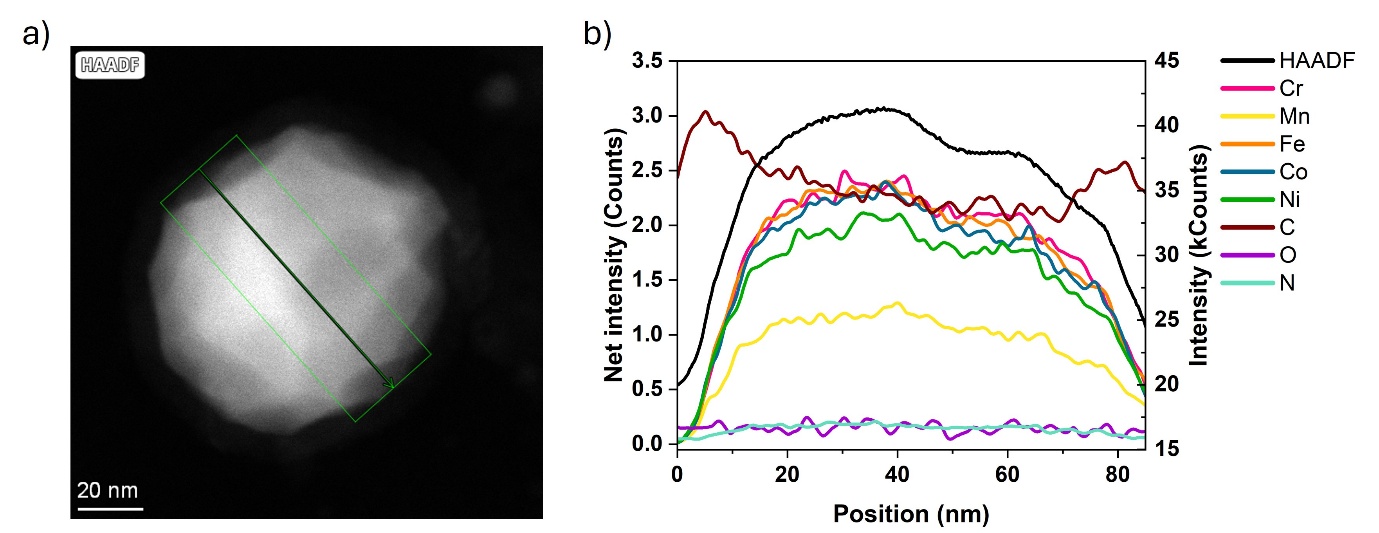


Figure S2 a) STEM-HAADF image of a selected HEA NP synthesized in acetonitrile and b) the corresponding EDS line profile showing strong C signal intensity and a very low intensity of O and N in the particle volume.


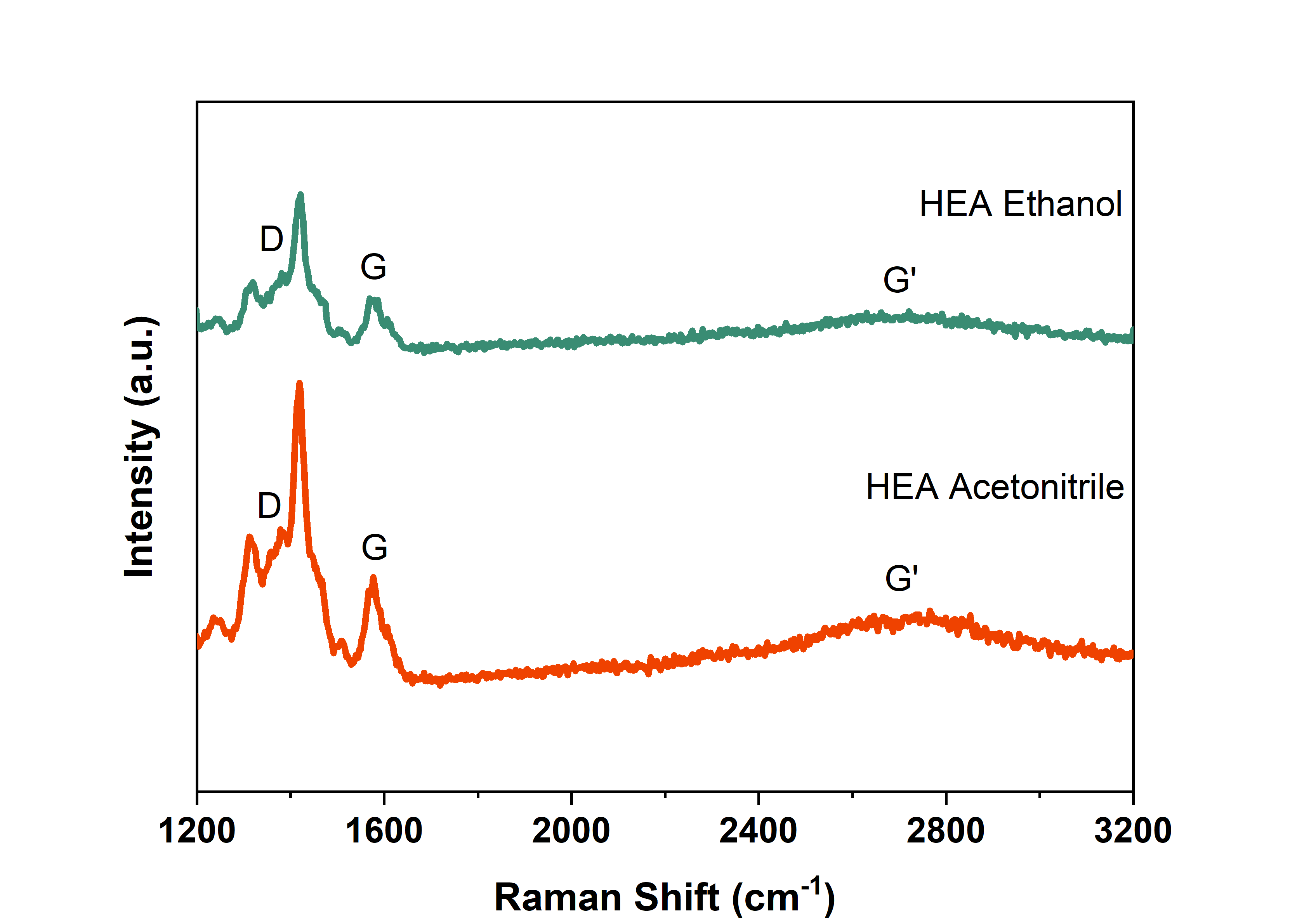


Figure S3 Raman spectra of the HEA NPs synthesized in acetonitrile and ethanol with marked regions of D, G, and G’ bands of graphitic carbon. The G-band (~1578 cm^-1^) corresponding to the graphitic sp^2^-carbon can be seen, while the position and intensity of the D-band (~1370 cm^-1^), representative of the structural disorder and defects in the graphitic shells, is shadowed by the neighbouring peaks [1–4]. Additionally, a shallow contribution from the G’ (or 2D) band can be seen around 2700 cm^-1^ [5].


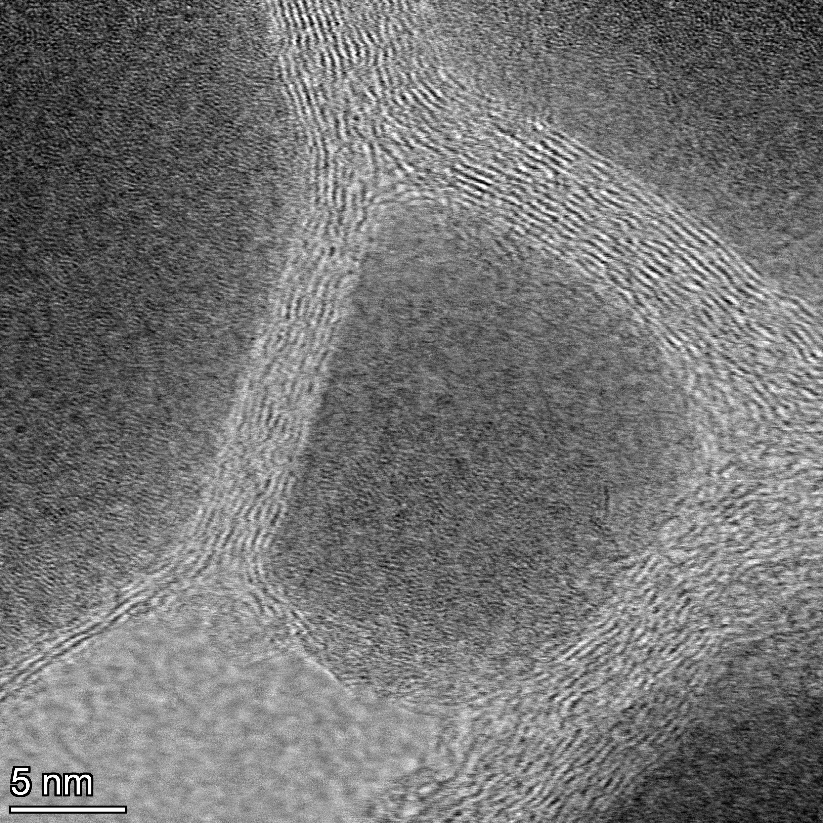


Figure S4 STEM bright field image highlighting the amorphous nature of a small HEA NP synthesized in acetonitrile surrounded by carbon shells.


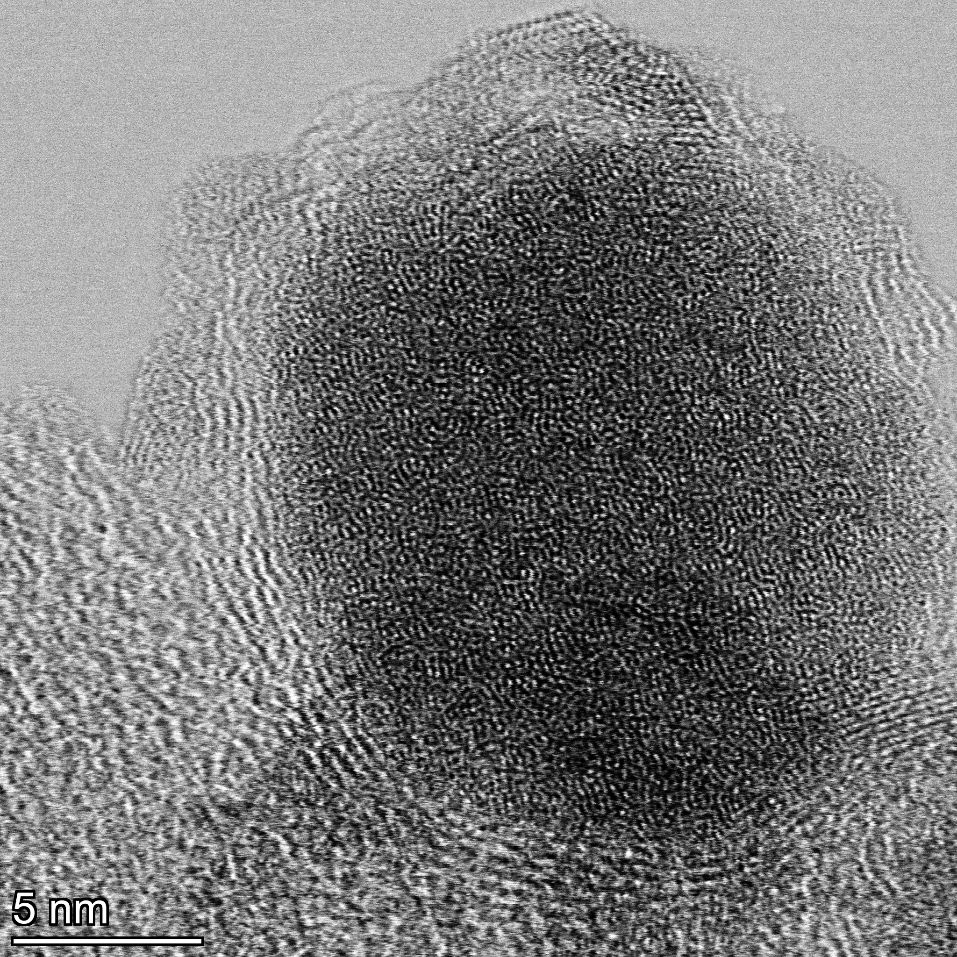


Figure S5 STEM bright field image highlighting the amorphous nature of a small HEA NP synthesized in acetonitrile surrounded by carbon shells.


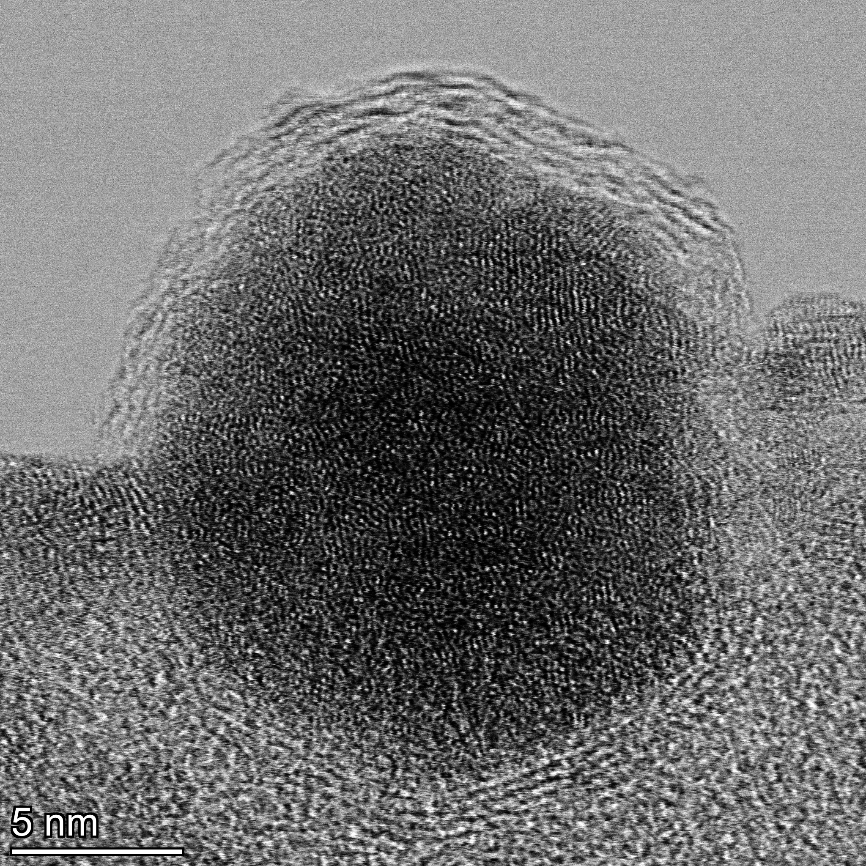


Figure S6 STEM bright field image highlighting the amorphous nature of a small HEA NP synthesized in acetonitrile surrounded by carbon shells.


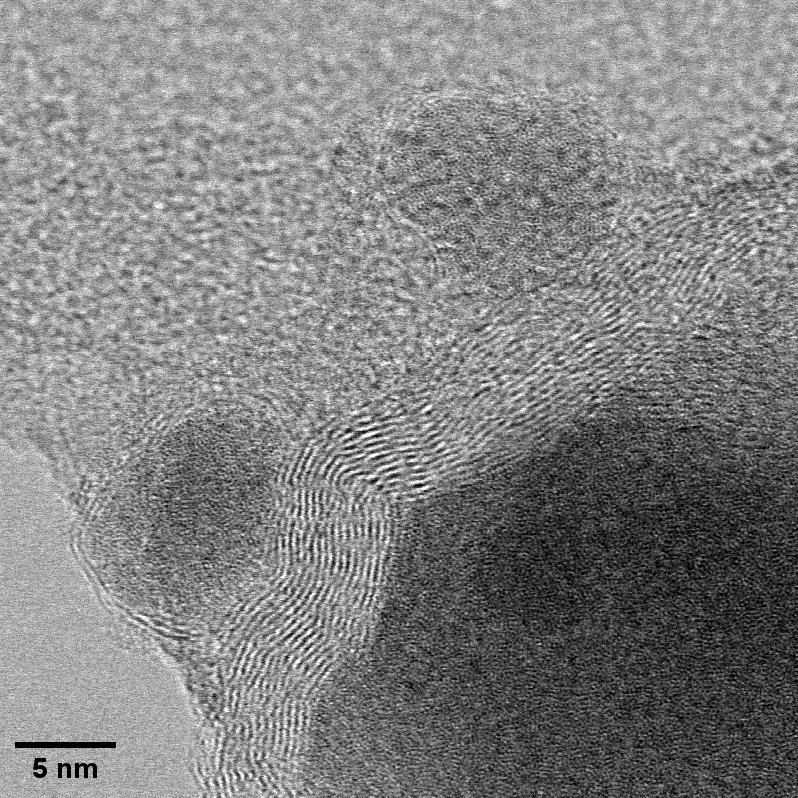


Figure S7 STEM bright field image highlighting the amorphous nature of HEA NPs synthesized in acetonitrile surrounded by carbon shells.


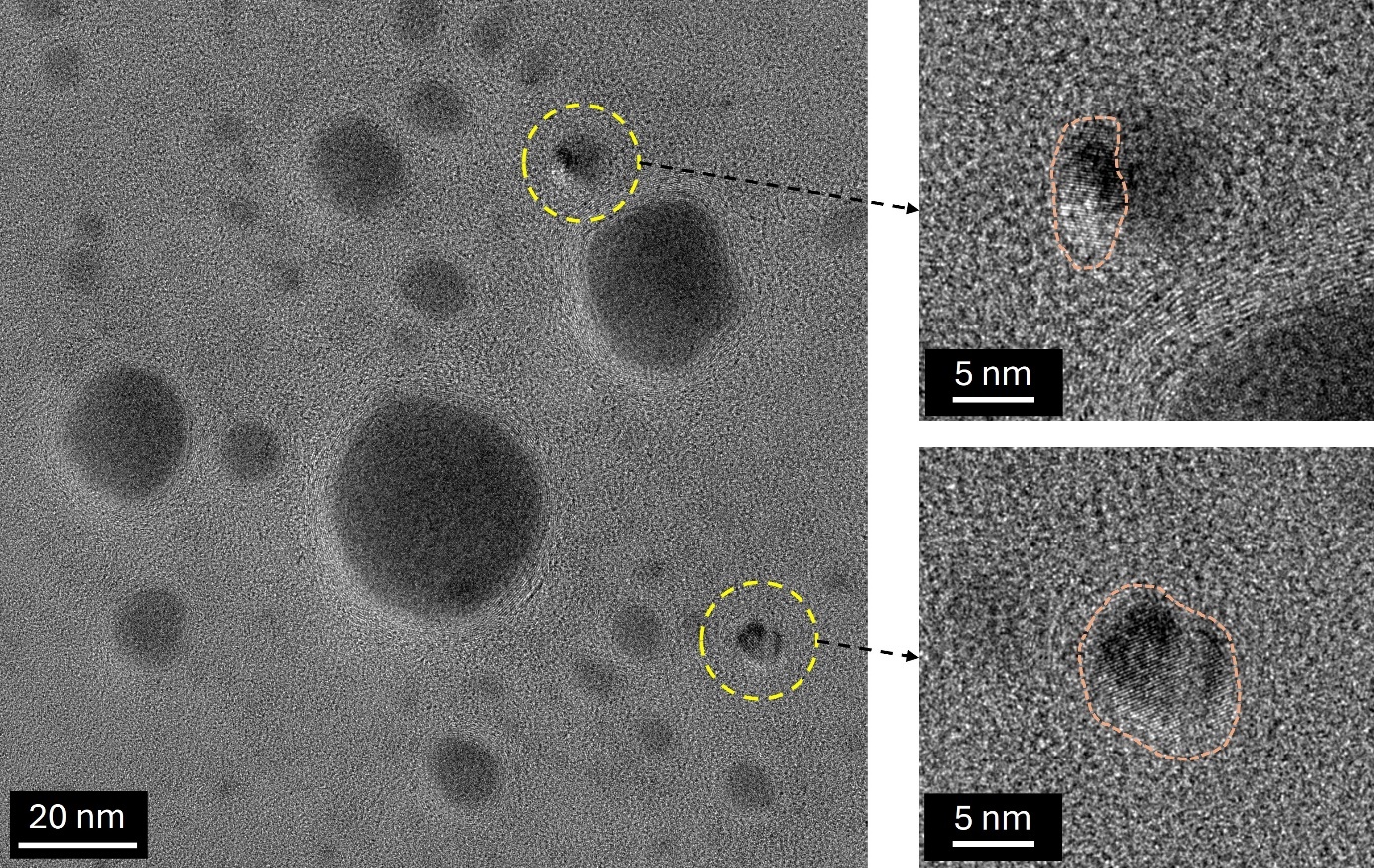


Figure S8 TEM bright field images showing selected NP regions from the HEA NPs synthesized in acetonitrile, highlighting the start of crystallization during the in-situ heating step of 350 to 400 °C.


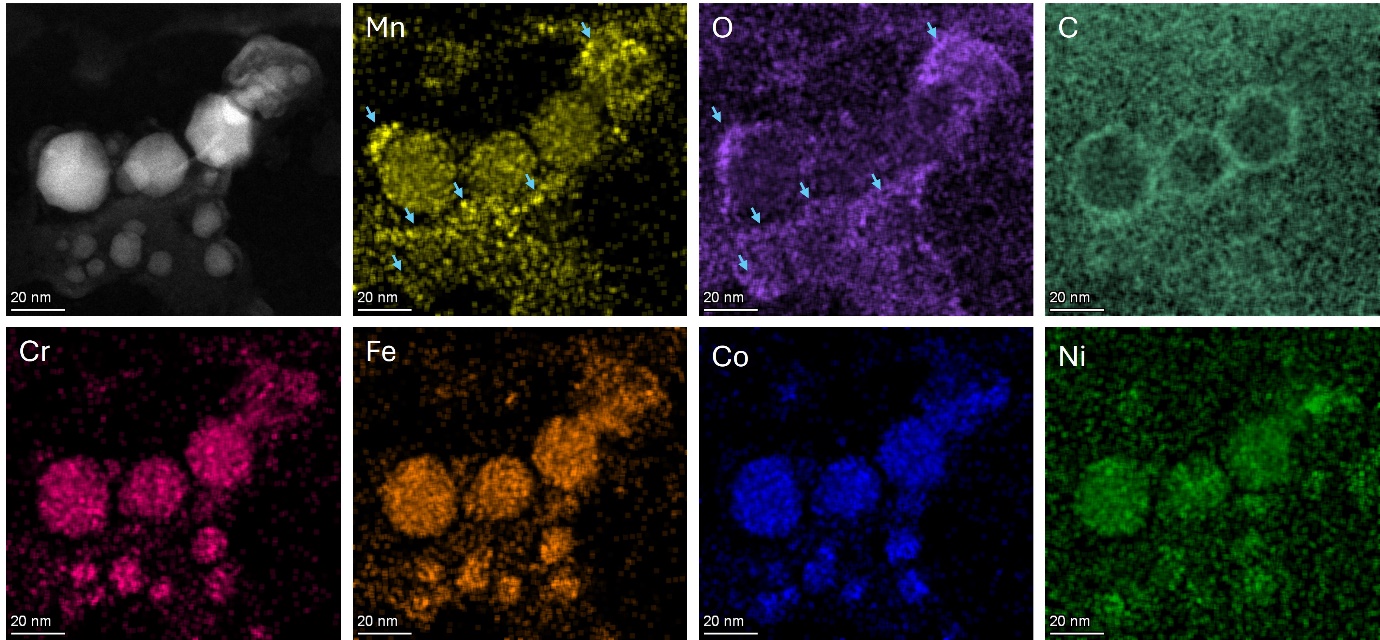


Figure S9 STEM-EDS mapping of HEA NPs synthesized in acetonitrile, highlighting Mn-rich fragments (blue arrows) outside the carbon shell, which appear to be oxidized post-ablation.


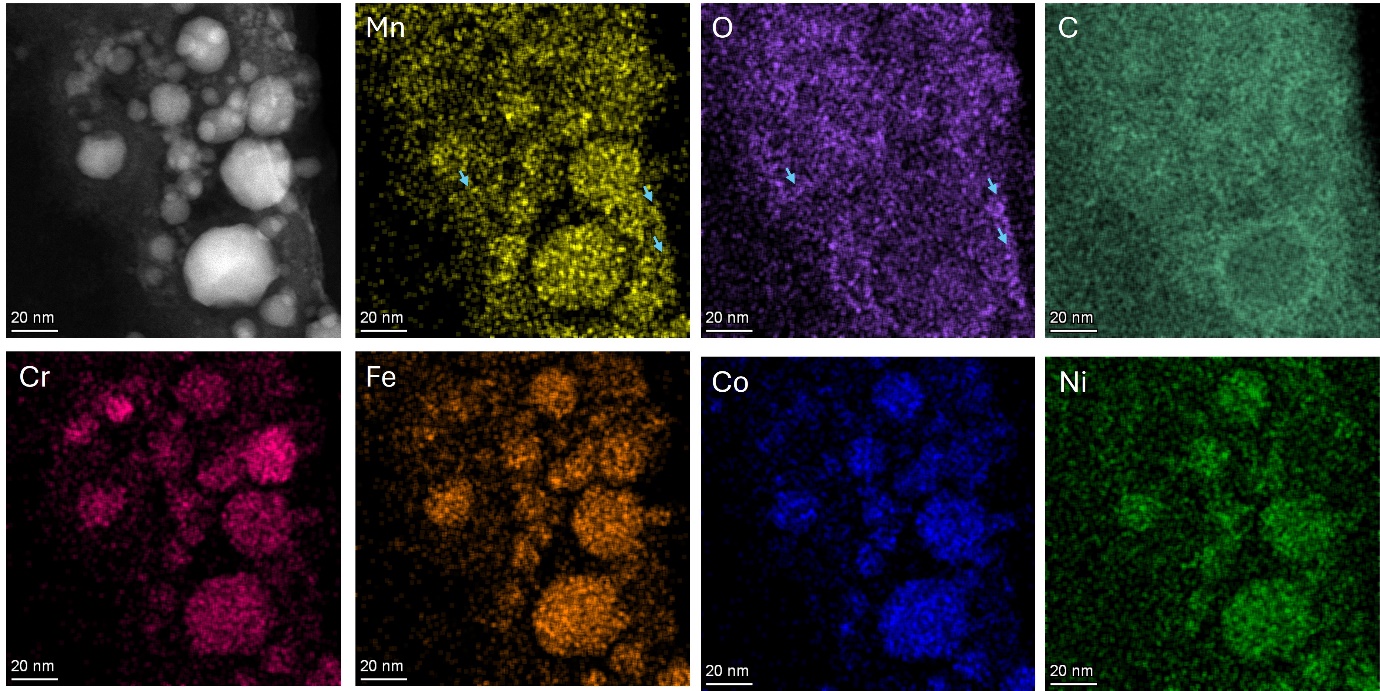


Figure S10 STEM-EDS mappings of HEA NPs synthesized in acetonitrile, highlighting Mn-rich fragments (blue arrows) outside the carbon shell, which appeared to be oxidized post-ablation.


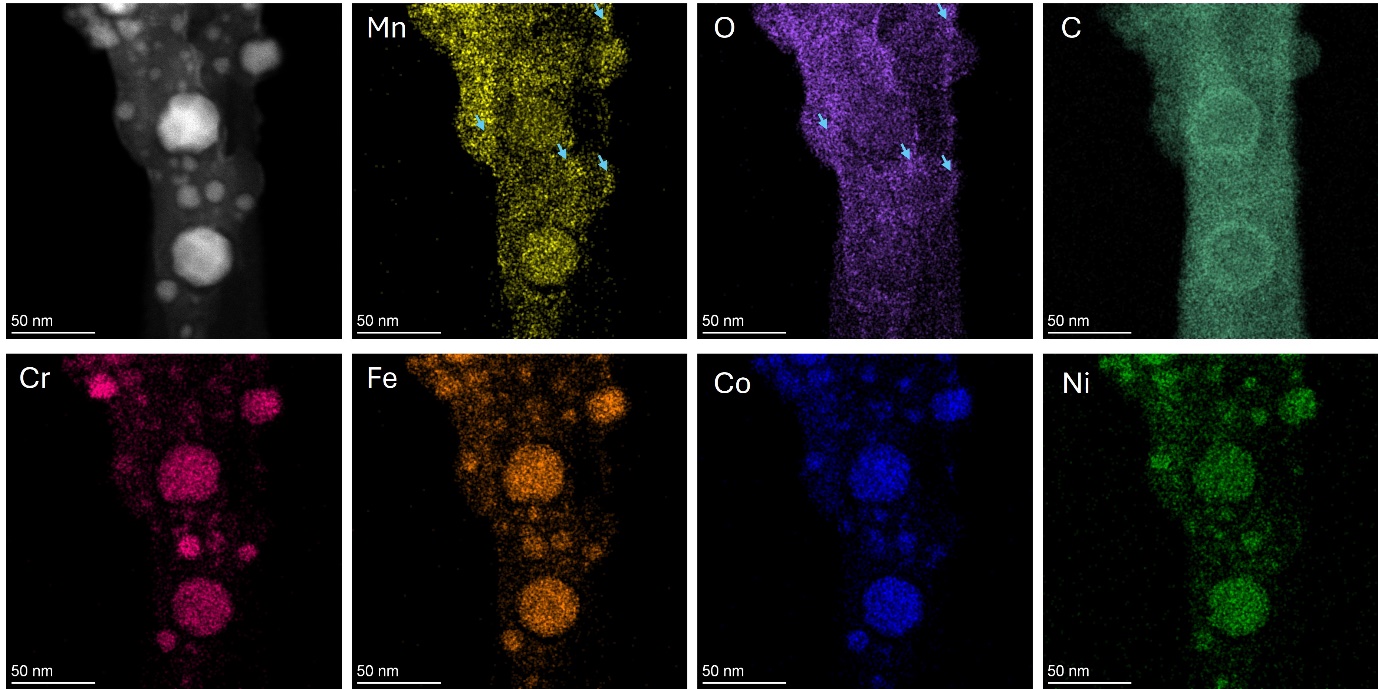


Figure S11 STEM-EDS mappings of HEA NPs synthesized in acetonitrile highlighting Mn-rich fragments (blue arrows) outside the carbon shell which appeared to be oxidized post-ablation.


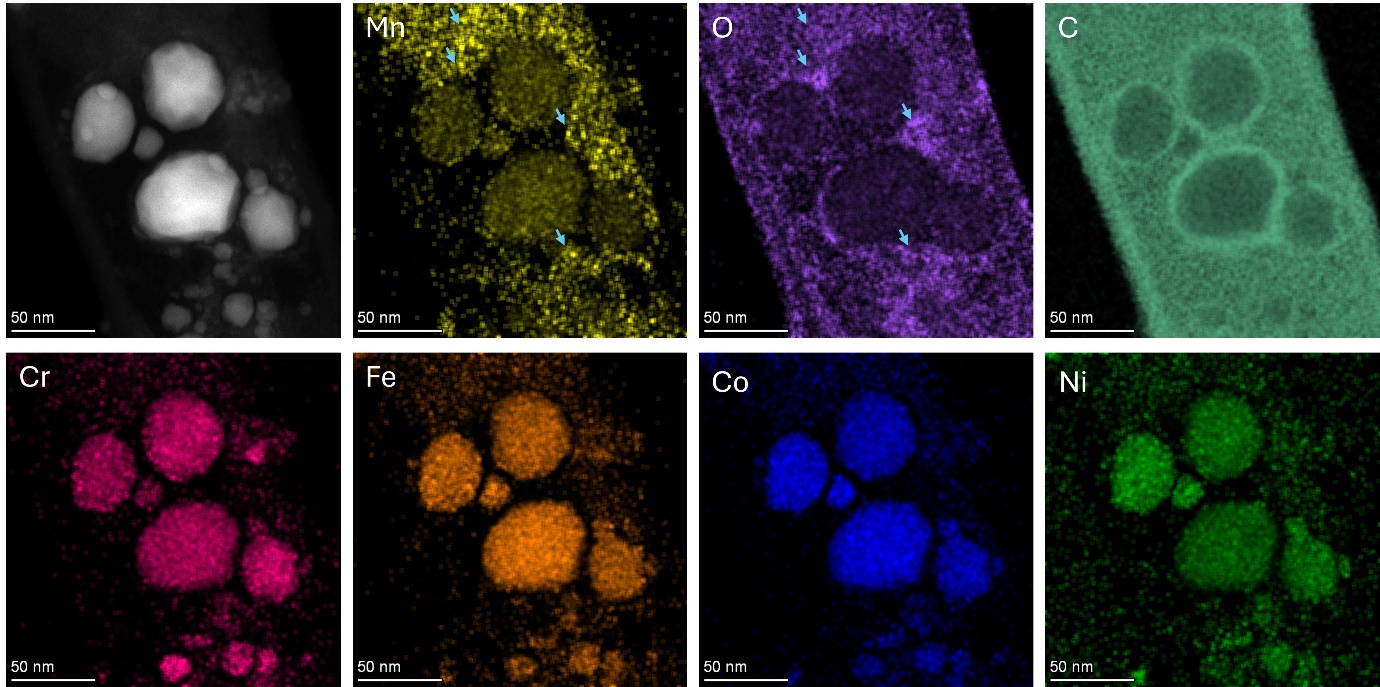


Figure S12 STEM-EDS mappings of HEA NPs synthesized in acetonitrile highlighting Mn-rich fragments (blue arrows) outside the carbon shell which appeared to be oxidized post-ablation.


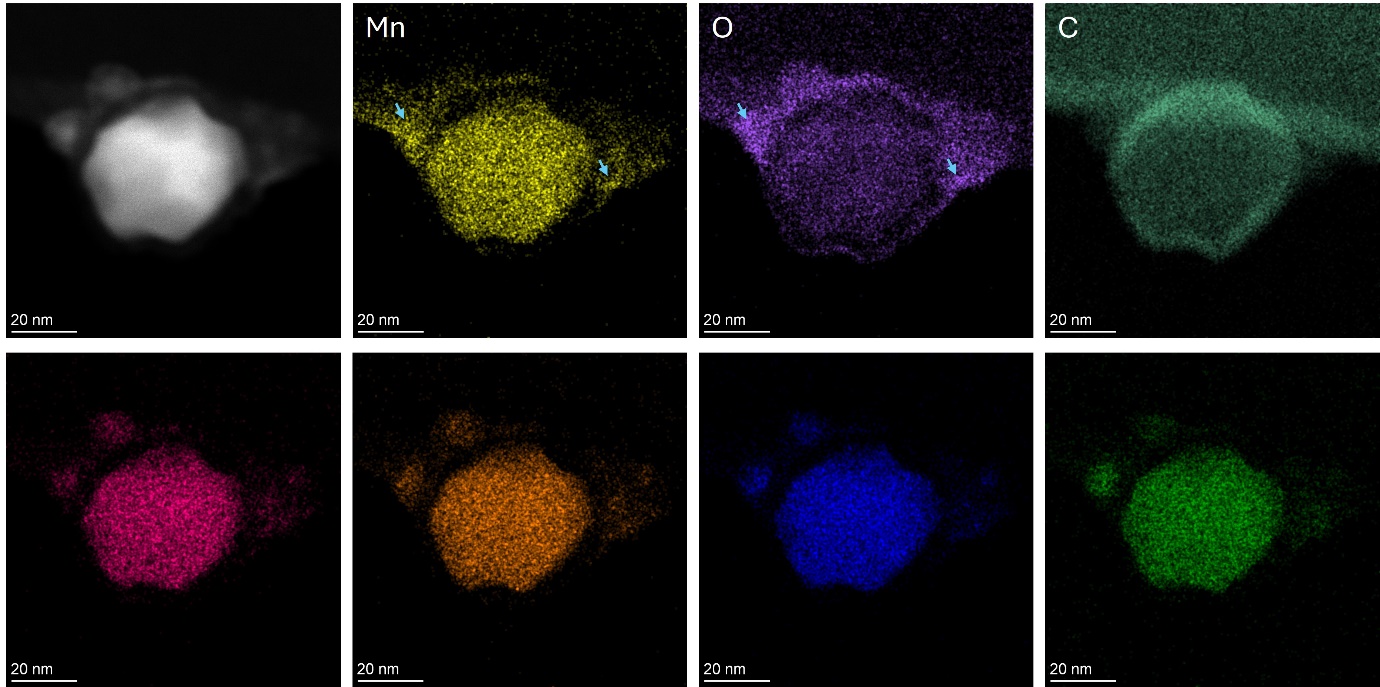


Figure S13 STEM-EDS mappings of HEA NPs synthesized in acetonitrile highlighting Mn-rich fragments (blue arrows) outside the carbon shell which appeared to be oxidized post-ablation.


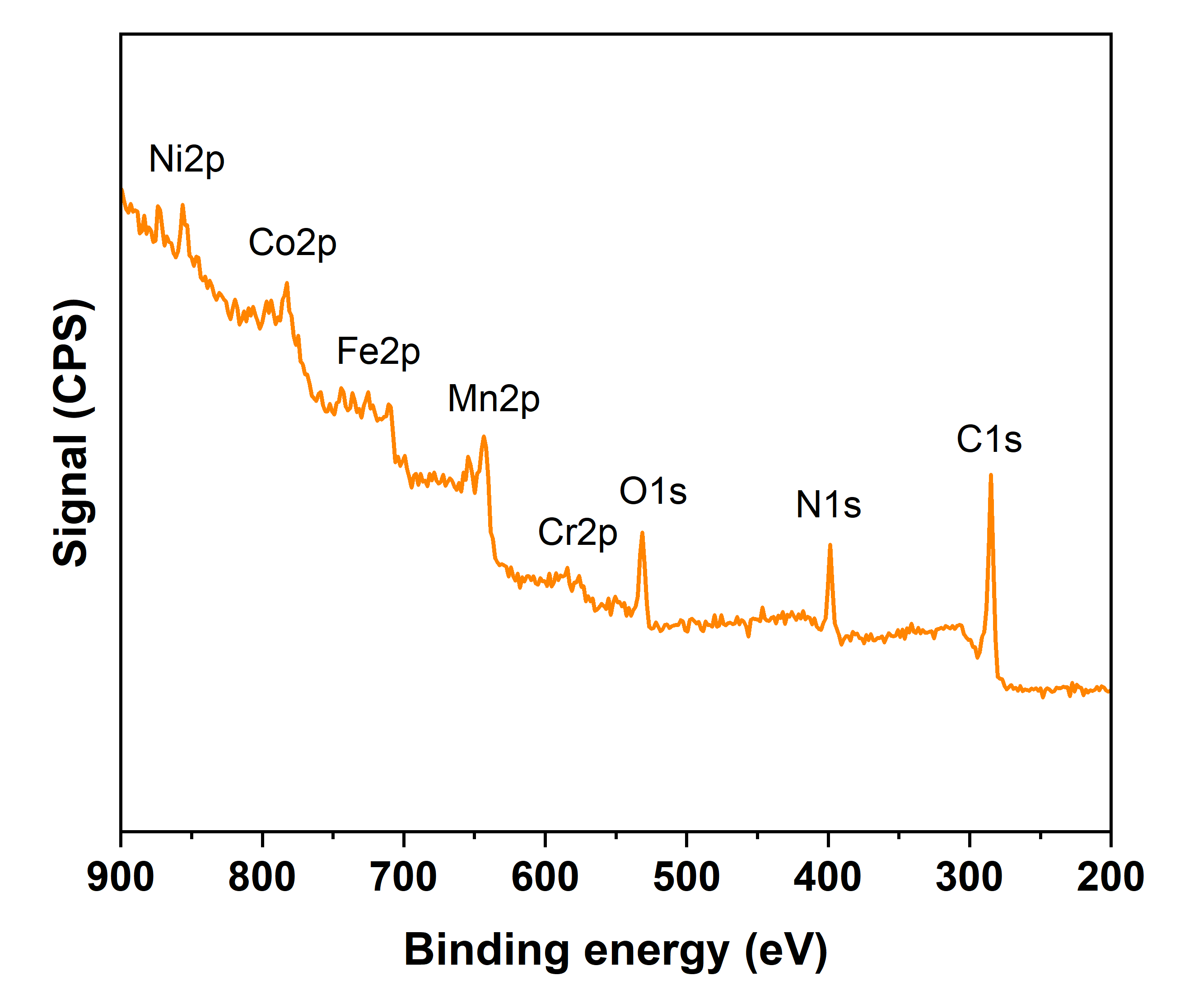


Figure S14 Exemplary XPS survey spectrum of HEA NPs synthesized in acetonitrile.

### Characteristics of nanoparticles in acetone

TEM characterization results performed for the NPs synthesized in acetone are shown in **Figure S9**. TEM and STEM bright field images (**Figures S9a – c**) reveal a nearly spherical morphology for larger particles, transitioning to rugged, for particles smaller than 40 nm. The particles display distinctive carbon shells, though these are less prominent compared to NPs synthesized in acetonitrile medium. This indicates a reduced carbon uptake during nanoparticle formation compared to the ablation process in acetonitrile, as the excess carbon is expelled to the NP surface for the formation of onion-like graphitic carbon shells. STEM-EDS mappings (**Figure S9d**) show a uniform distribution of all five elements, with pronounced carbon signals in shell regions and minimal oxygen content. SAED analysis (**Figures S9e and f**) confirms the amorphous nature of the nanoparticles regardless of size, with additional supporting SAED data in **Figure S10**. Despite the comparatively lower carbon uptake than in acetonitrile, the carbon content within the nanoparticles remains sufficient to stabilize the amorphous phase.


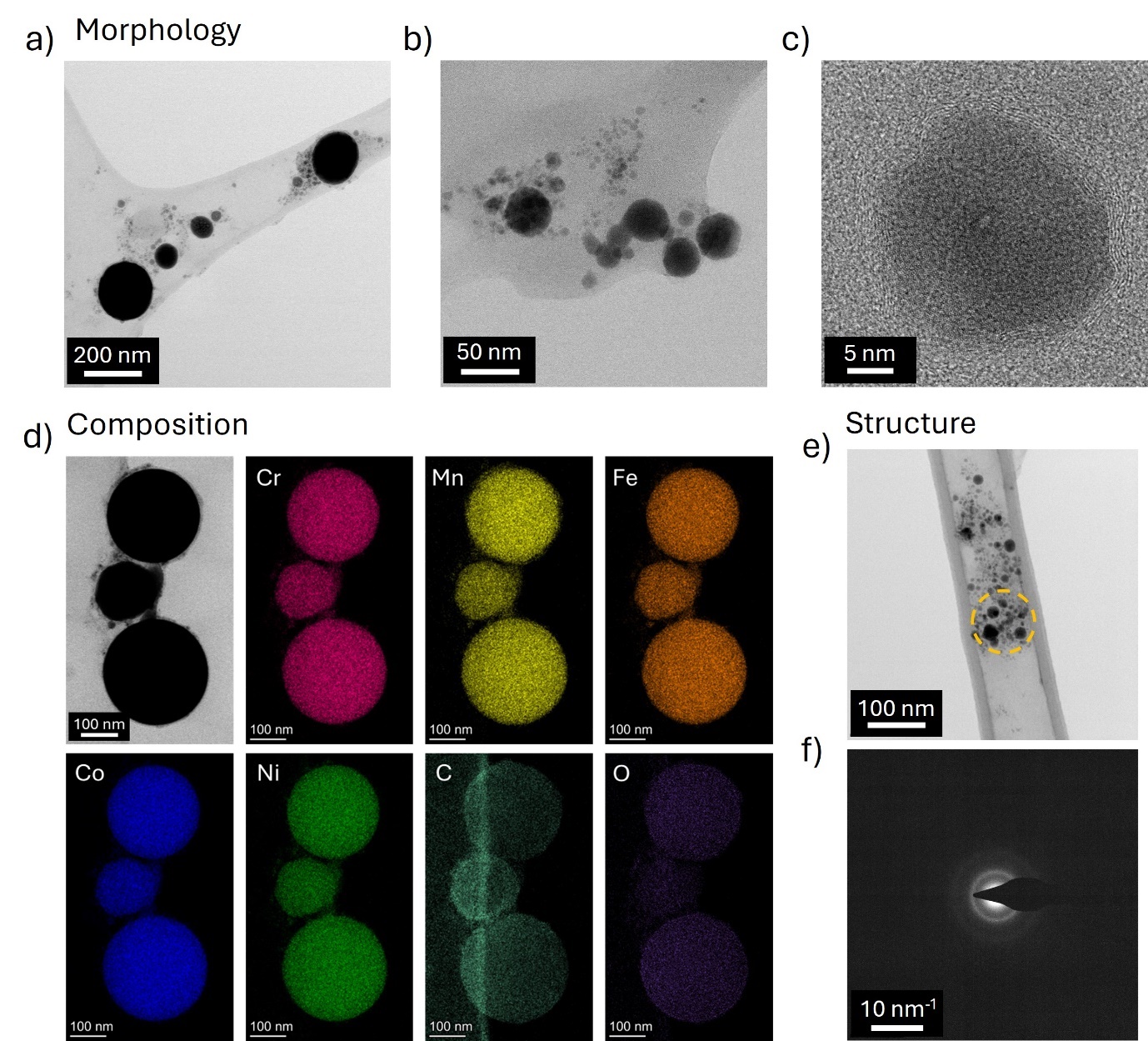


Figure S15 Morphology, composition and structural characterization of HEA NPs synthesized in acetone. a) TEM; b), c) STEM bright field images showing the nanoparticles’ morphology, size variations and carbon shell coverage; d) STEM-EDS analysis showing the individual element distribution maps; e), f) TEM bright field image and its respective SAED pattern highlighting the amorphous nature of the NPs.


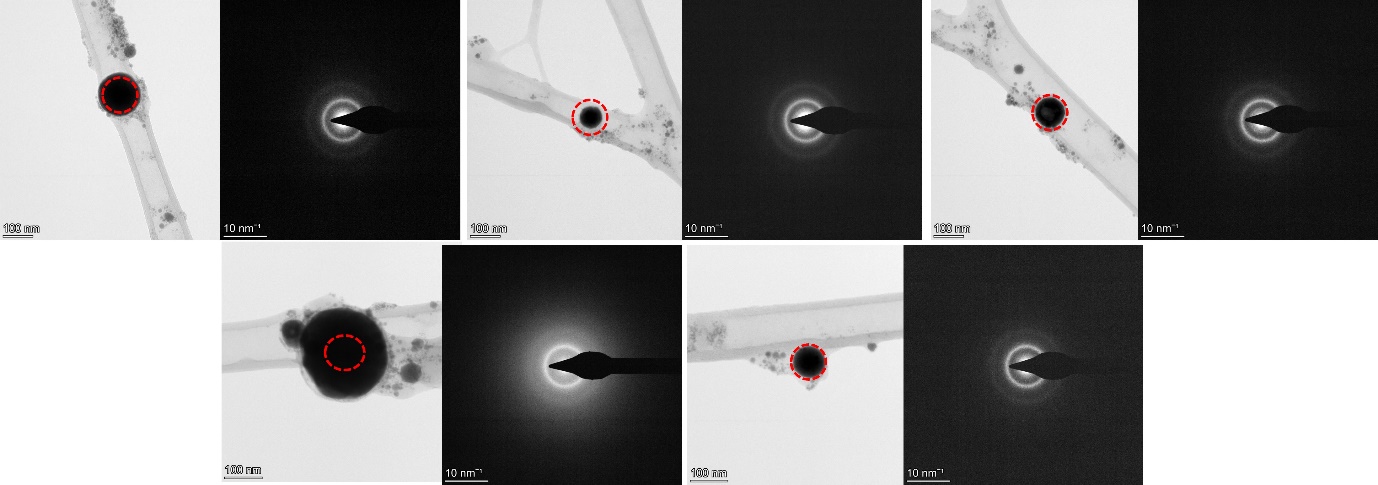


Figure S16 TEM bright field images and their corresponding SAED patterns of HEA NPs synthesized in acetone.

**Figures S11a and b** present XPS surface composition analysis for nanoparticles synthesized in the acetone medium with particle diameter, d, greater than 100 nm. Attempts to calculate the surface composition from other size sets were unsuccessful due to poor signal-to-noise ratios of the respective 2p and 3p spectra. Similar to acetonitrile samples, Mn enrichment is observed in the surface layers, though at a reduced level (approximately 34 at.% compared to 42 at.% in acetonitrile samples). Cr also shows sub-surface enrichment, evidenced by higher content in calculations from 3p peak deconvolution versus 2p peak analysis. Co and Ni appear to be distributed close to stoichiometric values in surface layers, while Fe is slightly lower at 16 at.%. **Figure S11c** depicts composition values from individual particles as a function of particle diameter measured using STEM-EDS. Unlike acetonitrile samples, individual nanoparticle volumes show less severe Mn depletion, with an average value of 16 ± 2.5 at.% Mn across all size ranges. No significant particle-size-dependent compositional deviations were observed for any of the other elements, with only minor concentration scatter for Mn and Fe in a few fine particles below 30 nm. Otherwise, Cr, Fe, Co, and Ni maintain near-stoichiometric distribution throughout the analyzed size range with average values of 19 ± 1.8 at.%, 21 ± 2.1 at.%, 21.6 ± 1.3 at.% and 22 ± 2.2 at.%, respectively.


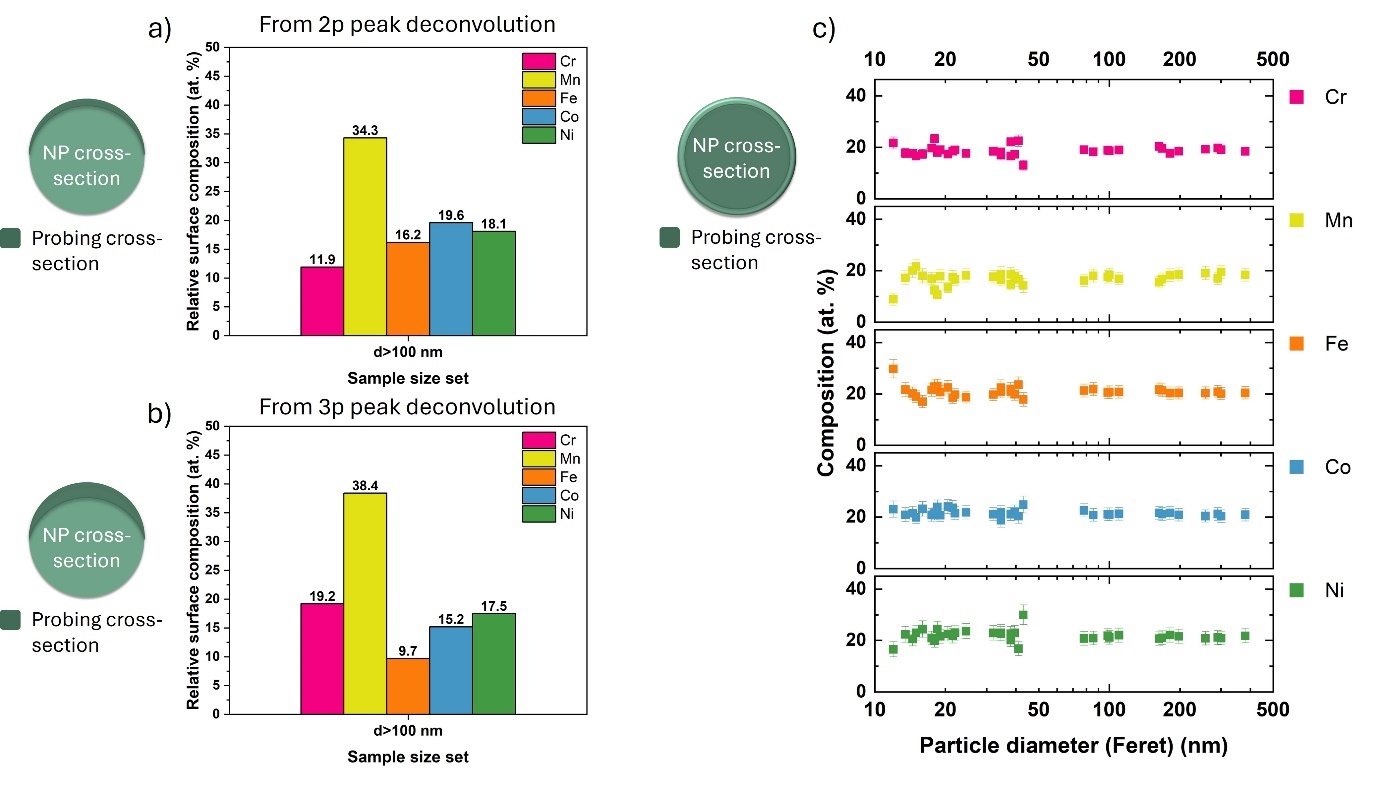


Figure S17 Size-dependent composition distributions in HEA NPs synthesized in acetone. a), b) Exemplary relative XPS surface elemental compositions of sub-set with a particle diameter greater than 100 nm calculated from the high-resolution XPS 2p and 3p spectra of the respective elements of the NPs synthesized in Acetone (Note: The signal intensity of the XPS spectra of other particle diameter sub-sets were not sufficient for the calculation of relative surface composition, hence omitted); c) Average elemental compositions as a function of particle diameter obtained from STEM-EDS of the NPs synthesized in acetone. The schematic representation on the left of the plots shows relative probing volume cross-sections compared to the NP cross-sections of the respective measurement variant.

### Characteristics of nanoparticles in ethanol

**Figure S12** presents the morphology, composition, and structural characterization of HEA nanoparticles synthesized in ethanol. Most particles exhibit s spherical morphology, with rugged surfaces observed only in smaller particles below 15 nm diameter (**Figures S12a-c**). Particle surfaces display thin carbon shells that are less prominent compared to those in acetone and acetonitrile samples (**Figure S13**). STEM-EDS mappings reveal a uniform distribution of all elements with reduced carbon and oxygen signal intensity (**Figure S12d**). TEM-SAED analysis indicates primarily amorphous structures (**Figures S12e and f**), while powder-XRD analysis (**Figure S12g**) shows low-intensity (111) and (200) reflections of the fcc phase alongside the amorphous structure, suggesting partial crystallization in the NPs.


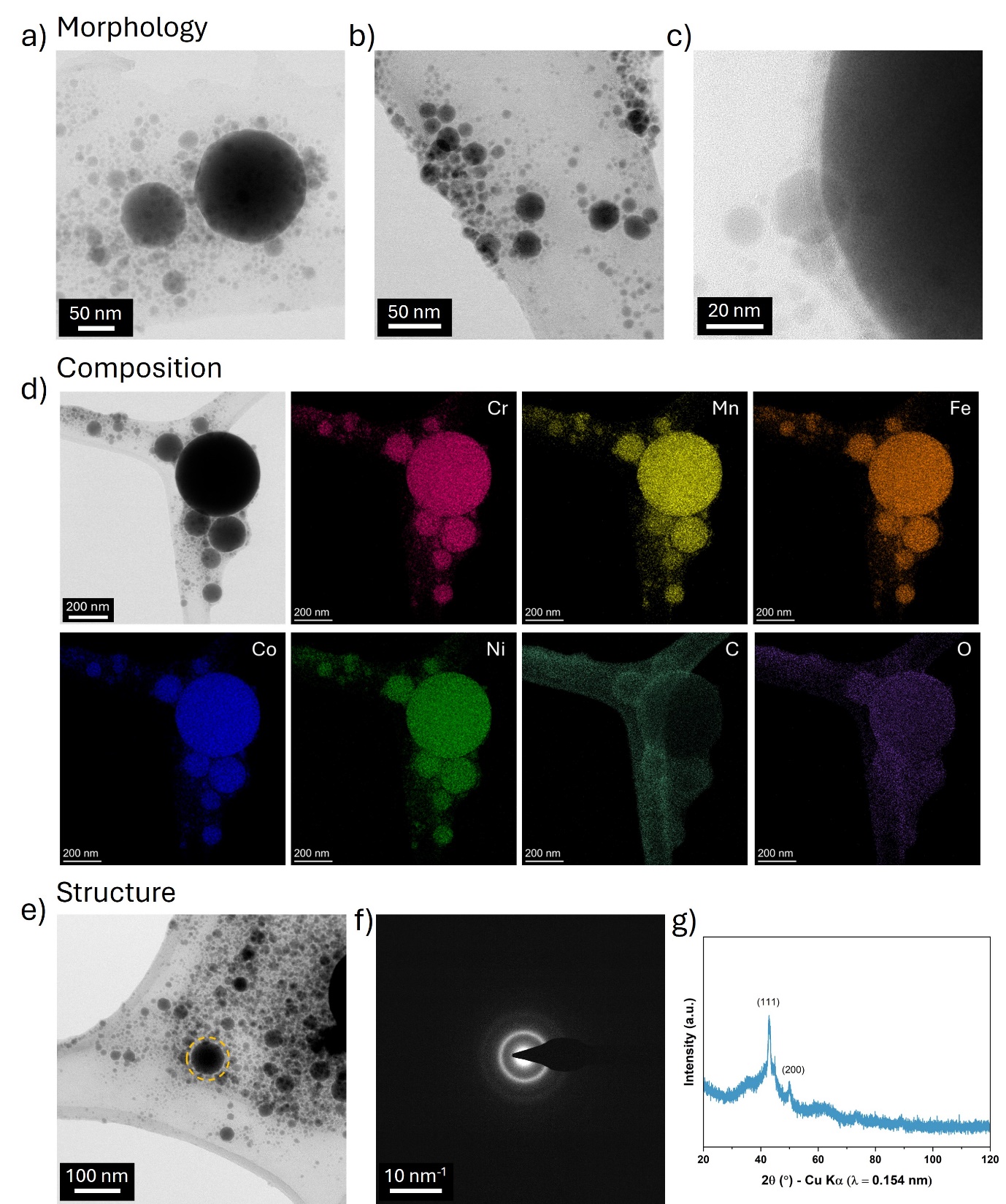


Figure S18 Morphology, composition, and structural characterization of HEA NPs synthesized in ethanol. a), b), c) STEM bright field images showing the nanoparticles' morphology, size variations and carbon shell coverage; d) STEM-EDS analysis showing the individual element distribution maps; e), f) TEM bright field image and its respective SAED pattern highlighting the amorphous nature of the NPs; g) Powder XRD pattern of the NPs showing a broad diffused peak highlighting their amorphous nature with minor reflections indicating the presence of a small fraction of fcc solid solution phase.


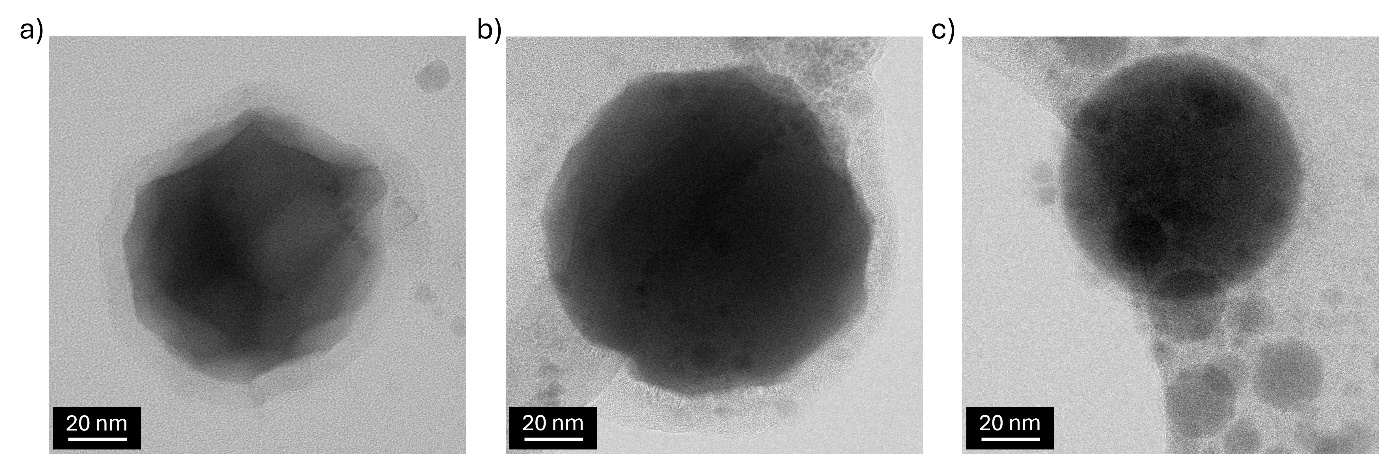


Figure S19 STEM bright field images highlighting the differences in morphology and carbon shell thickness of HEA NPs synthesized in (a) acetonitrile, (b) acetone and (c) ethanol, respectively.

The composition analysis of the HEA NPs synthesized in ethanol, conducted using XPS and STEM-EDS, is presented in **Figure S14**. Surface composition calculations derived from 2p and 3p peak deconvolutions across different size sets reveal Mn enrichment (**Figures S14a and b**), with a concentration of approximately 32 at.%, compared to 34 at.% in acetone samples and 42 at.% in acetonitrile samples. As observed in previous cases, sub-surface Cr enrichment is evident in the ethanol samples, while Fe, Ni, and Co contents follow trends similar to those observed in acetone samples. **Figure S14c** illustrates average elemental composition values for individual particles as a function of particle diameter, determined via STEM-EDS. Fe, Co, and Ni exhibit uniform distributions throughout the analyzed particle size range, with average values slightly exceeding the expected stoichiometry: 21.3 ± 1.2 at.%, 21.6 ± 0.9 at.%, and 21.4 ± 1.2 at.%, respectively. Comparable to the results in acetonitrile, Mn loss within the particle volume is minimal, demonstrating near-uniform distribution across the size range. However, deviations in the concentration trend are observed for Mn in particles smaller than 30 nm in diameter, with corresponding minor adjustments in Cr and Fe concentrations. For particles exceeding 30 nm in diameter, the average Mn concentration is approximately 16 ± 1.9 at.%, while for particles below 30 nm, the value is around 17 ± 3.1 at.%. The average Cr concentration across all size ranges is 19.6 ± 1.6 at.%.


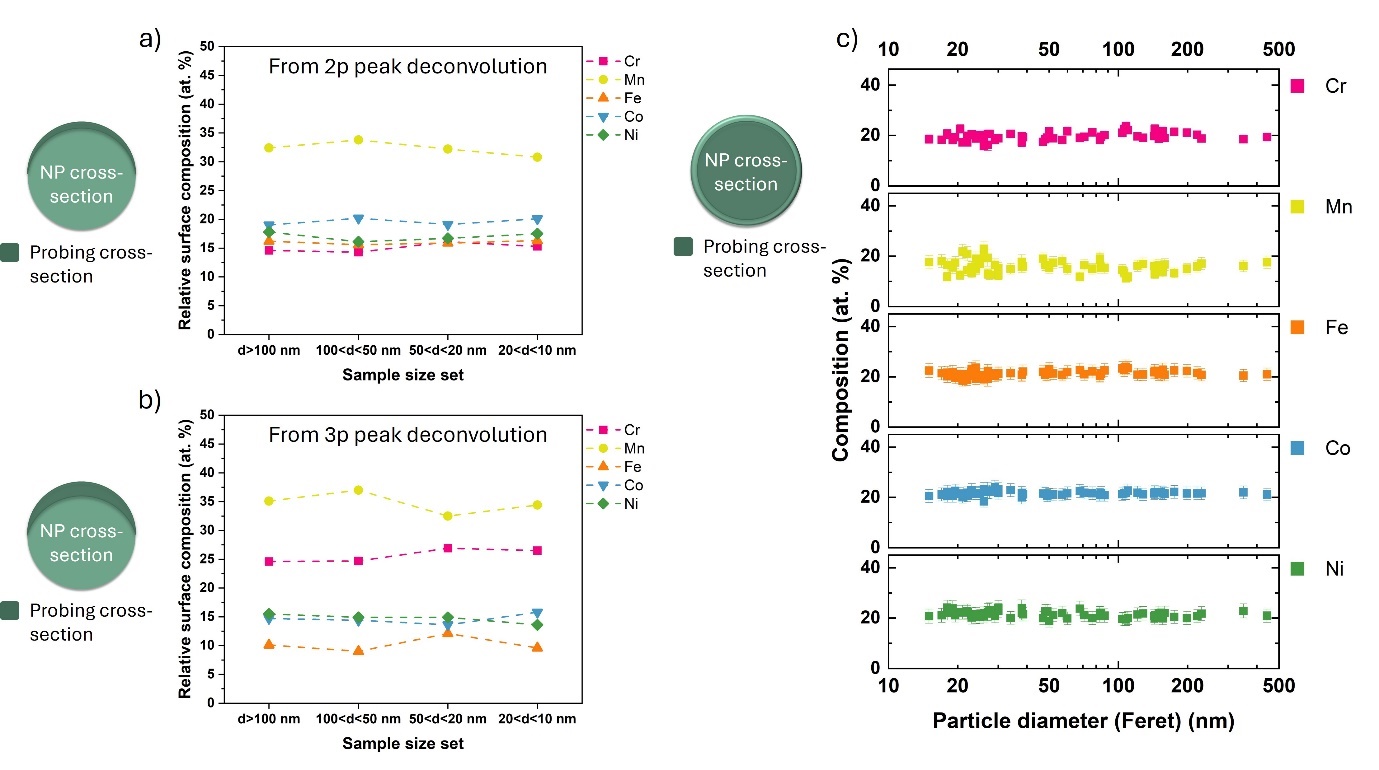


Figure S20 Size-dependent composition distributions in HEA NPs synthesized in ethanol. a), b) Relative XPS surface elemental compositions as a function of different particle diameter sub-sets calculated from the high-resolution XPS 2p and 3p spectra of the respective elements of the NPs synthesized in Ethanol (Note: The signal intensity of the XPS spectra of the particle diameter sub-set less than 10 nm were not sufficient for the calculation of relative surface composition, hence omitted); c) Average elemental compositions as a function of particle diameter obtained from STEM-EDS of the NPs synthesized in ethanol. The schematic representation on the left of the plots shows relative probing volume cross-sections compared to the NP cross-sections of the respective measurement variant.

### Target characterization

XRD and SEM-EDS analysis were used to evaluate the phase structure and macroscopic elemental concentrations of the CrMnFeCoNi target material. **Figure S15a** shows the XRD pattern of the target, indicating the peaks corresponding to a single-phase fcc crystal structure. **Figure S15b** shows the SEM-EDS mapping of a selected region on the polished target surface, where no macroscopic element segregation was found. APT compositional analysis was performed to ensure the uniform elemental mixing at the nanoscale, as preferential segregation in the target material would affect the resulting nanoparticle compositions. 3-dimensional (3D) atom maps of the constituent elements shown in **Figure S15c** show a uniform distribution, within the limits of resolution of APT [6,7]. 1D compositional analysis using a 45 nm-diameter cylindrical region of interest (ROI) from a selected region is shown in **Figure S15d,** highlighting the near-equimolar and uniform distribution of the constituents. A spider plot with the average elemental compositions of the target material obtained from SEM-EDS and APT compositional analysis is shown in **Figure S15e** and the respective values are listed in **Table S1.**


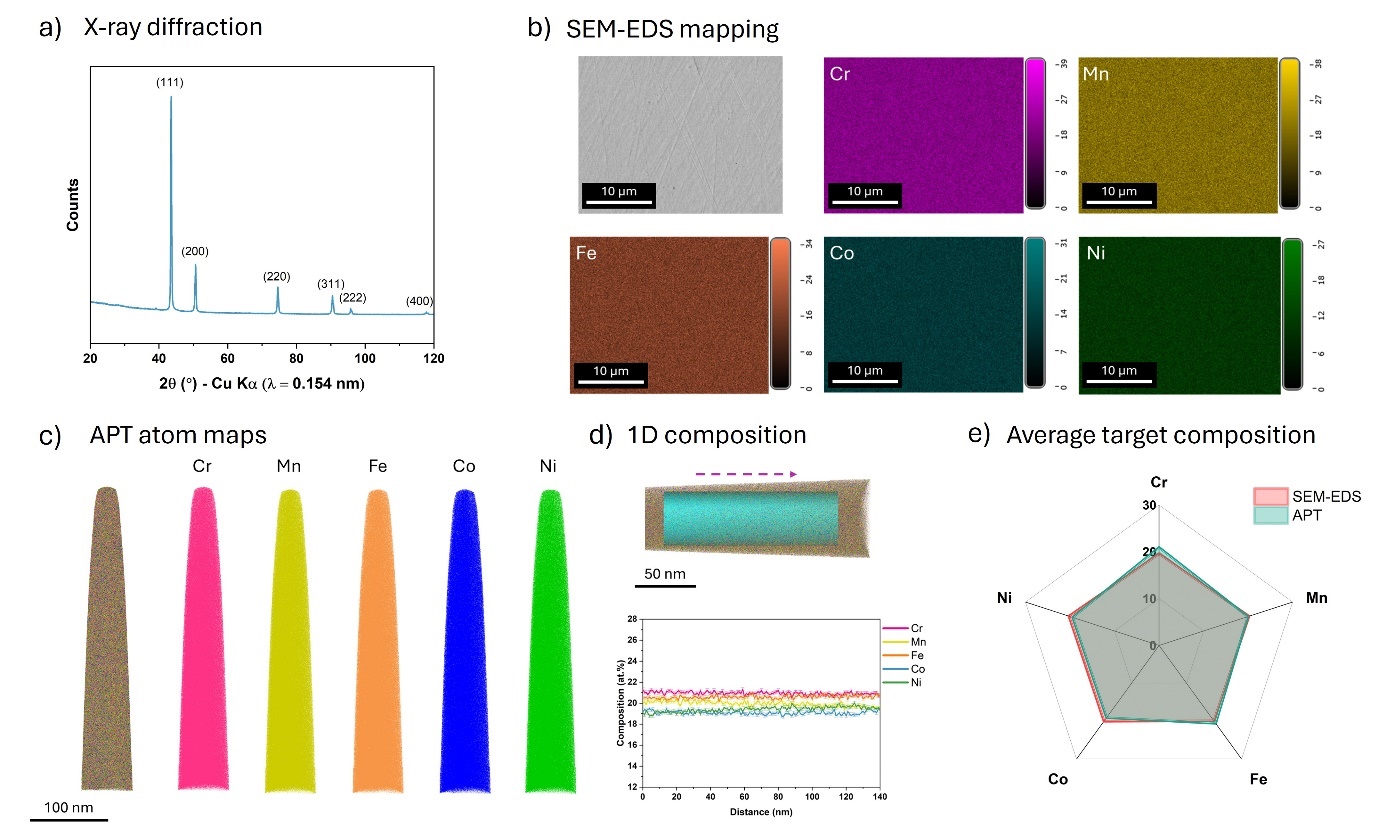


Figure S21 Structural and compositional characterization of the CrMnFeCoNi bulk target. a) XRD pattern of the polished bulk target showing peaks corresponding to a single phase fcc crystal structure; b) SEM-EDS mapping of individual elements over a selected region on the polished bulk target surface; c) 3D APT reconstruction with individual element distribution maps of the bulk target showing no element segregations at the nanoscale; d) 1D concentration profile of individual elements from a selected region of the 3D reconstruction highlighting the near-equimolar stoichiometry of the target at the nanoscale; e) Spider plot showing the average elemental compositions of the target material obtained from SEM-EDS and APT compositional analysis.

Table S1 Average elemental concentrations from SEM-EDS and APT measurements of the CrMnFeCoNi bulk target.

| Element | Average composition from  SEM-EDS | | Average composition from APT | |
| --- | --- | --- | --- | --- |
|  | Atomic % | Error % | Atomic % | Error % |
| Cr | 19.59 | 2.9 | 20.95 | 0.15 |
| Mn | 20.22 | 2.9 | 19.95 | 0.14 |
| Fe | 19.91 | 3.1 | 20.59 | 0.15 |
| Co | 20.03 | 3.1 | 19.09 | 0.14 |
| Ni | 20.25 | 3.4 | 19.42 | 0.14 |

### XPS characterization

Table S2 Constraints used during the peak fitting of XPS 2p spectra listed for each element.

| Cr 2p | Cr^0^ | | Cr(III) oxide (5 multiplets) | | Cr(III) hydroxide | | Cr(VI) oxide | |
| --- | --- | --- | --- | --- | --- | --- | --- | --- |
|  | min | Max | min | max | min | max | min | max |
| Pos. Constr. / eV | 574 | 574.4 | 575.5 | 579.1 | 577.1 | 577.5 | 479.4 | 479.8 |
| FWHM Constr. / eV | 0.7 | 1.1 | 0.8 | 1 | 2.5 | 2.7 | 1.28 | 1.48 |
|  |  |  |  |  |  |  |  |  |
| Mn 2p | Mn^0^ | | MnO (6 multiplets) | | MnOOH (5 multiplets) | | MnO_2_ (5 multiplets) | |
|  | min | Max | min | max | min | max | min | max |
| Pos. Constr. / eV | 638.5 | 639 | 640.2 | 645.9 | 641.1 | 645.5 | 642.4 | 646.8 |
| FWHM Constr. / eV | 1 | 1.3 | 1.5 | 1.95 | 1.05 | 1.45 | 1.05 | 1.45 |
|  |  |  |  |  |  |  |  |  |
| Fe 2p | Fe^0^ | | FeO (5 multiplets) | | Fe_2_O_3_ (6 multiplets) | | FeOOH (6 multiplets) | |
|  | min | max | min | max | min | max | min | max |
| Pos. Constr. / eV | 706.4 | 708 | 708.2 | 716.8 | 709.6 | 720.8 | 710.1 | 721.1 |
| FWHM Constr. / eV | 0.6 | 1 | 1.1 | 2.7 | 0.9 | 2.8 | 1.2 | 2.7 |
|  |  |  |  |  |  |  |  |  |
| Co 2p | Co^0^  (3 multiplets) | | CoO  (4 multiplets) | | Co(OH)_2_  (4 multiplets) | | CoOOH  (4 multiplets) | |
|  | min | max | min | max | min | max | min | max |
| Pos. Constr. / eV | 777.7 | 783.5 | 779.6 | 786.9 | 780 | 790.8 | 779.7 | 790.57 |
| FWHM Constr. / eV | 0.5; 2.8 | 0.9; 3.2 | 2.4 | 2.8 | 2.2 | 2.8 | 1.28 | 1.68 |
|  |  |  |  |  |  |  |  |  |
| Co 2p |  |  |  |  |  |  | Co_3_O_4_  (5 multiplets) | |
|  |  |  |  |  |  |  | min | max |
| Pos. Constr. / eV |  |  |  |  |  |  | 779.2 | 789.9 |
| FWHM Constr. / eV |  |  |  |  |  |  | 1.1 | 1.7 |
|  |  |  |  |  |  |  |  |  |
| Ni 2p | Ni^0^  (3 multiplets) | | NiO  (5 multiplets) | | Ni(OH)_2_  (6 multiplets) | |  |  |
|  | min | max | min | max | min | max |  |  |
| Pos. Constr. / eV | 851.6 | 859.8 | 852.7 | 867.5 | 853.9 | 867.6 |  |  |
| FWHM Constr. / eV | 0.9; 2.28 | 1.1; 2.68 | 0.9; 3.1 | 1.1; 4.2 | 1.4; 2.85 | 1.7; 3.25 |  |  |
|  |  |  |  |  |  |  |  |  |

Table S3 Constraints used during the peak fitting of XPS 3p spectra listed for each element.

|  | Cr 3p | | Mn 3p | | Fe 3p | | Co 3p | |
| --- | --- | --- | --- | --- | --- | --- | --- | --- |
|  | min | max | min | max | min | max | min | max |
| Pos. Constr. / eV | 46 | 47 | 47 | 50.4 | 53.7 | 56.7 | 59.9 | 62.5 |
| FWHM Constr. / eV | 0.6 | 5 | 0.6 | 5 | 0.6 | 5 | 0.6 | 5 |
|  |  |  |  |  |  |  |  |  |
|  | Ni 3p | |  |  |  |  |  |  |
|  | min | max |  |  |  |  |  |  |
| Pos. Constr. / eV | 67.5 | 69 |  |  |  |  |  |  |
| FWHM Constr. / eV | 0.6 | 5 |  |  |  |  |  |  |


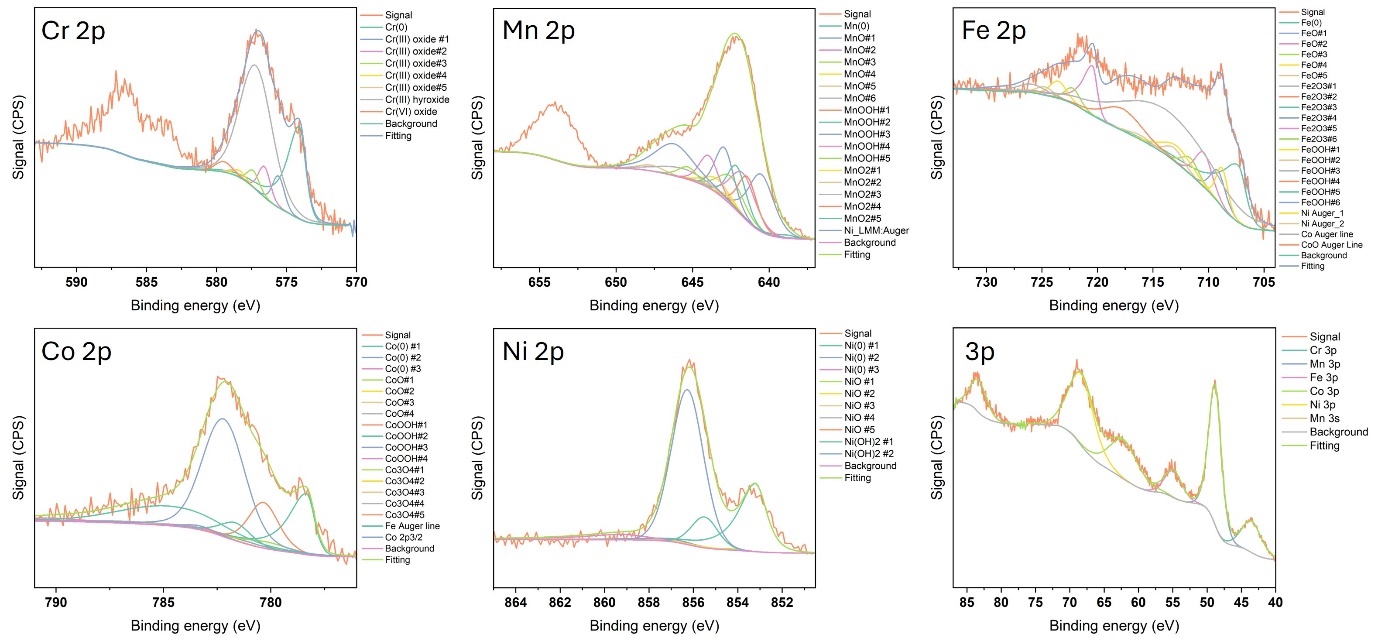


Figure S22 Deconvoluted Cr 2p, Mn 2p, Fe 2p, Co 2p, Ni 2p and 3p XPS spectra of HEA NPs synthesized in acetonitrile within the size set d > 100 nm.


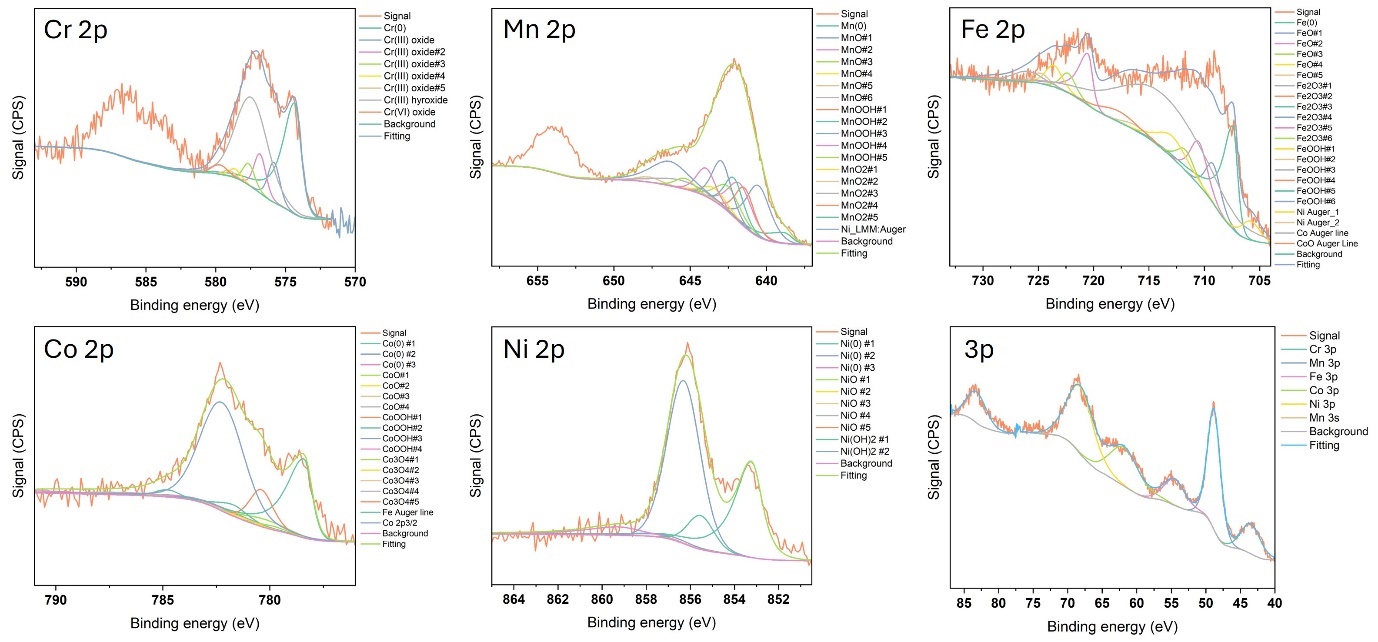


Figure S23 Deconvoluted Cr 2p, Mn 2p, Fe 2p, Co 2p, Ni 2p and 3p XPS spectra of HEA NPs synthesized in acetonitrile within the size set 100nm < d < 50 nm.


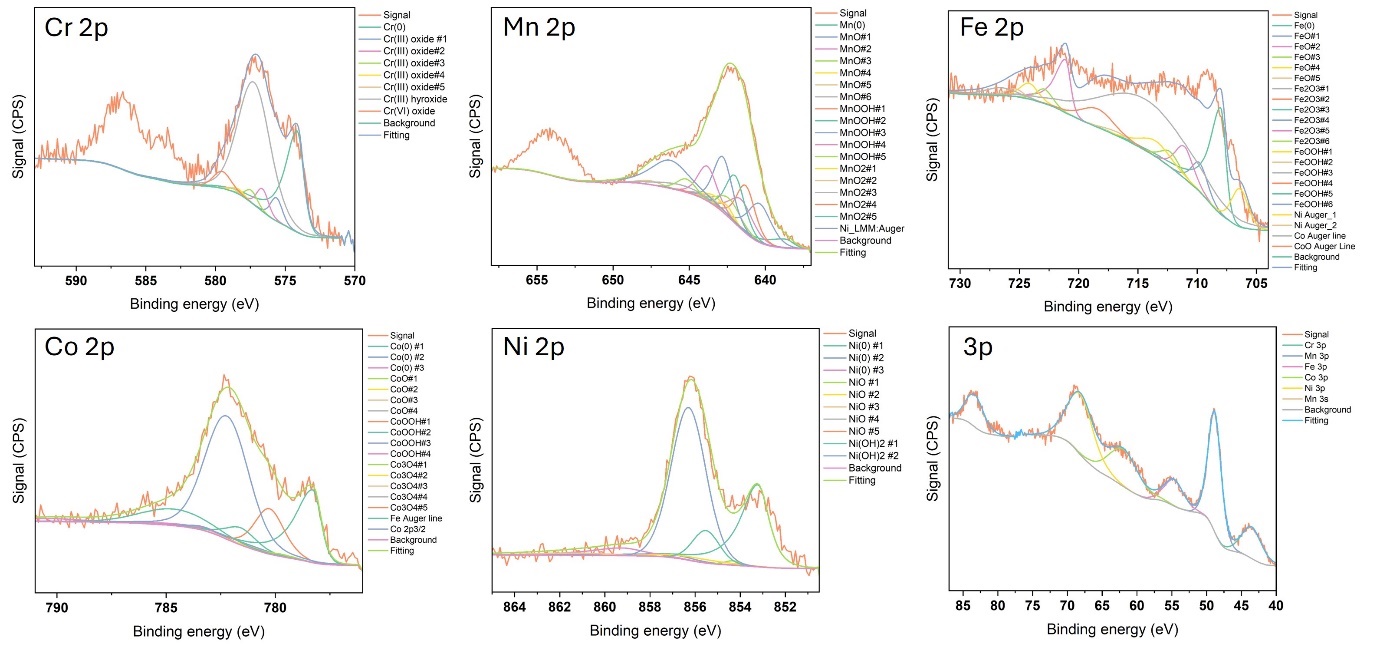


Figure S24 Deconvoluted Cr 2p, Mn 2p, Fe 2p, Co 2p, Ni 2p and 3p XPS spectra of HEA NPs synthesized in acetonitrile within the size set 50nm < d < 20 nm.


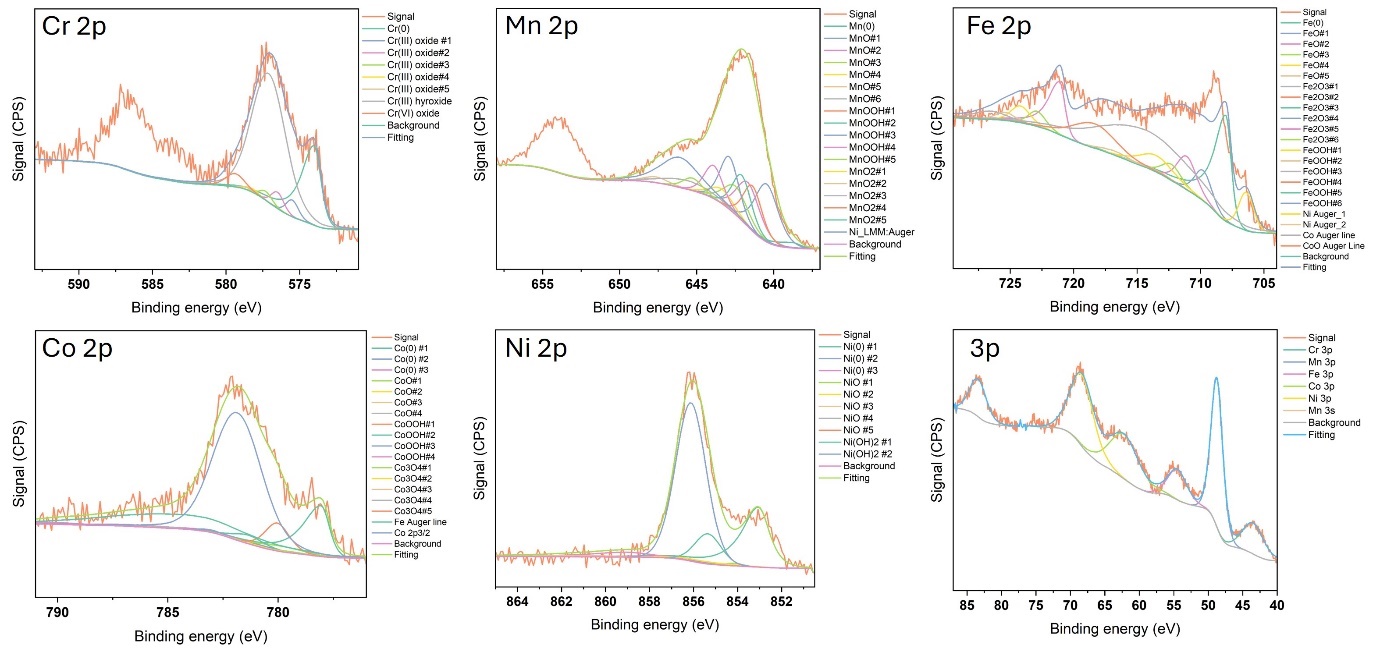


Figure S25 Deconvoluted Cr 2p, Mn 2p, Fe 2p, Co 2p, Ni 2p and 3p XPS spectra of HEA NPs synthesized in acetonitrile within the size set 20nm < d < 10 nm.


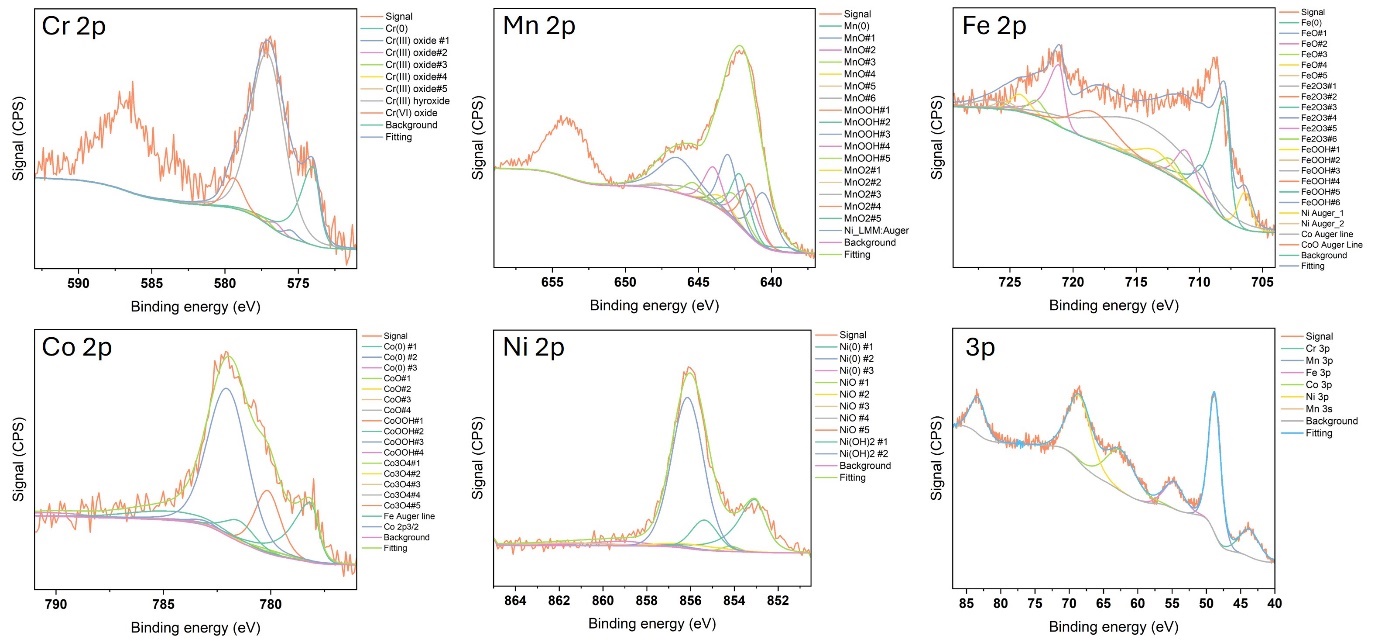


Figure S26 Deconvoluted Cr 2p, Mn 2p, Fe 2p, Co 2p, Ni 2p and 3p XPS spectra of HEA NPs synthesized in acetonitrile within the size set d < 10 nm.


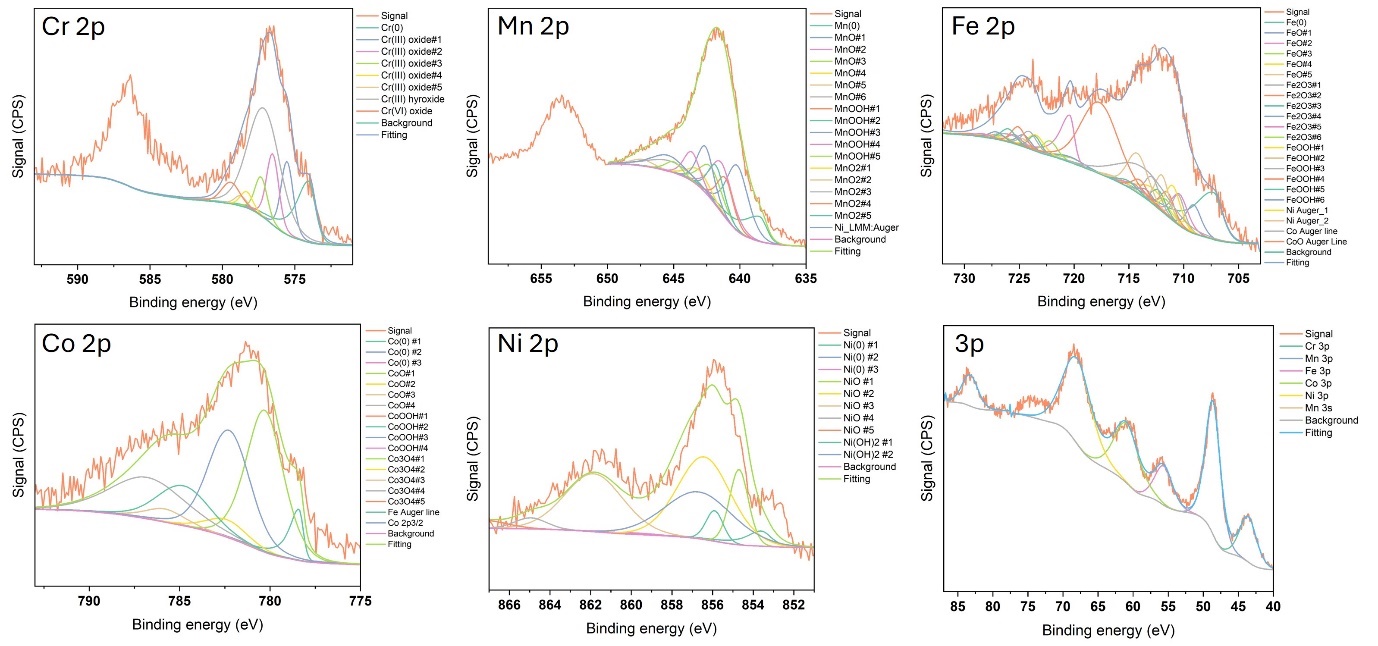


Figure S27 Deconvoluted Cr 2p, Mn 2p, Fe 2p, Co 2p, Ni 2p and 3p XPS spectra of HEA NPs synthesized in acetone within the size set d > 100 nm.


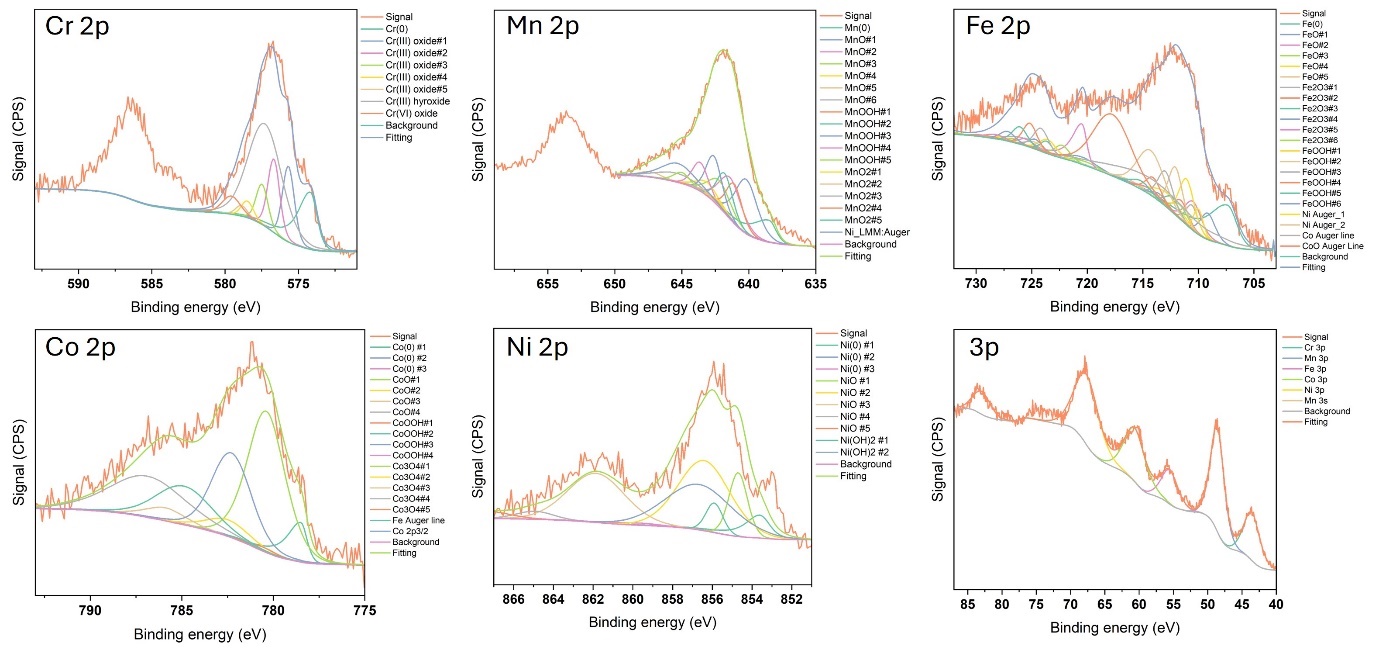


Figure S28 Deconvoluted Cr 2p, Mn 2p, Fe 2p, Co 2p, Ni 2p and 3p XPS spectra of HEA NPs synthesized in ethanol within the size set d > 100 nm.


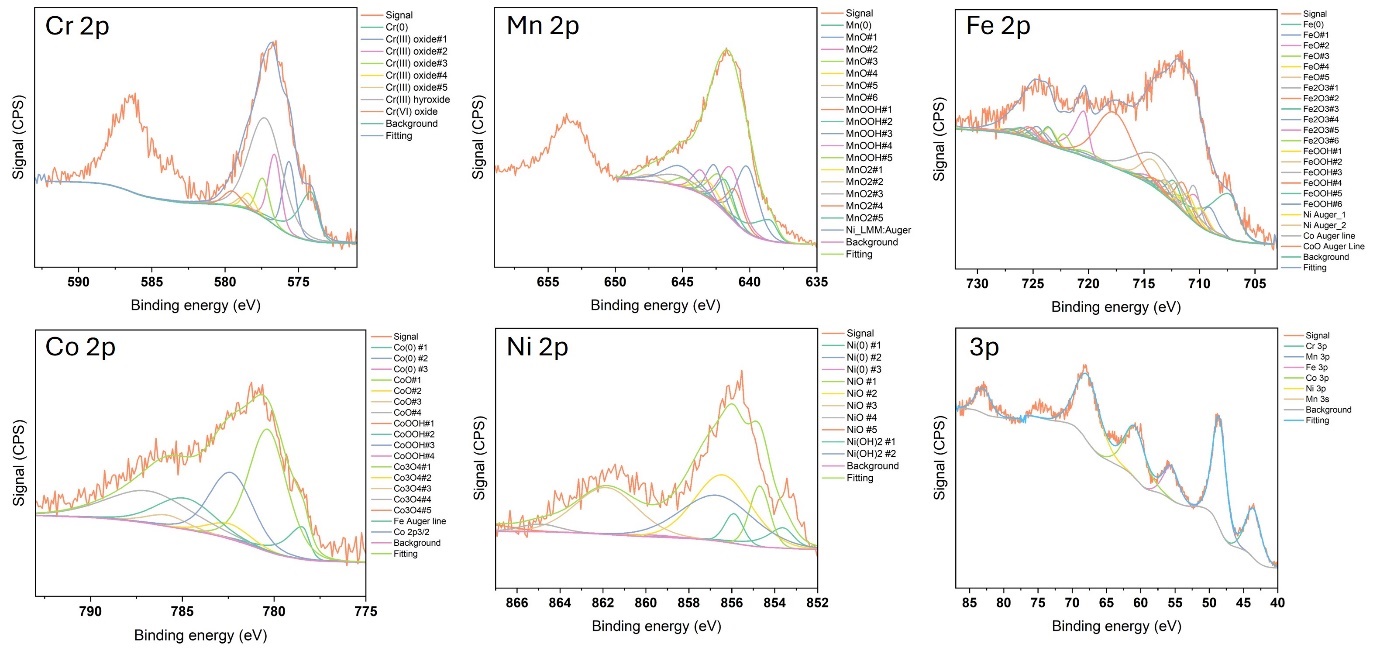


Figure S29 Deconvoluted Cr 2p, Mn 2p, Fe 2p, Co 2p, Ni 2p and 3p XPS spectra of HEA NPs synthesized in ethanol within the size set 100 nm < d < 50 nm.


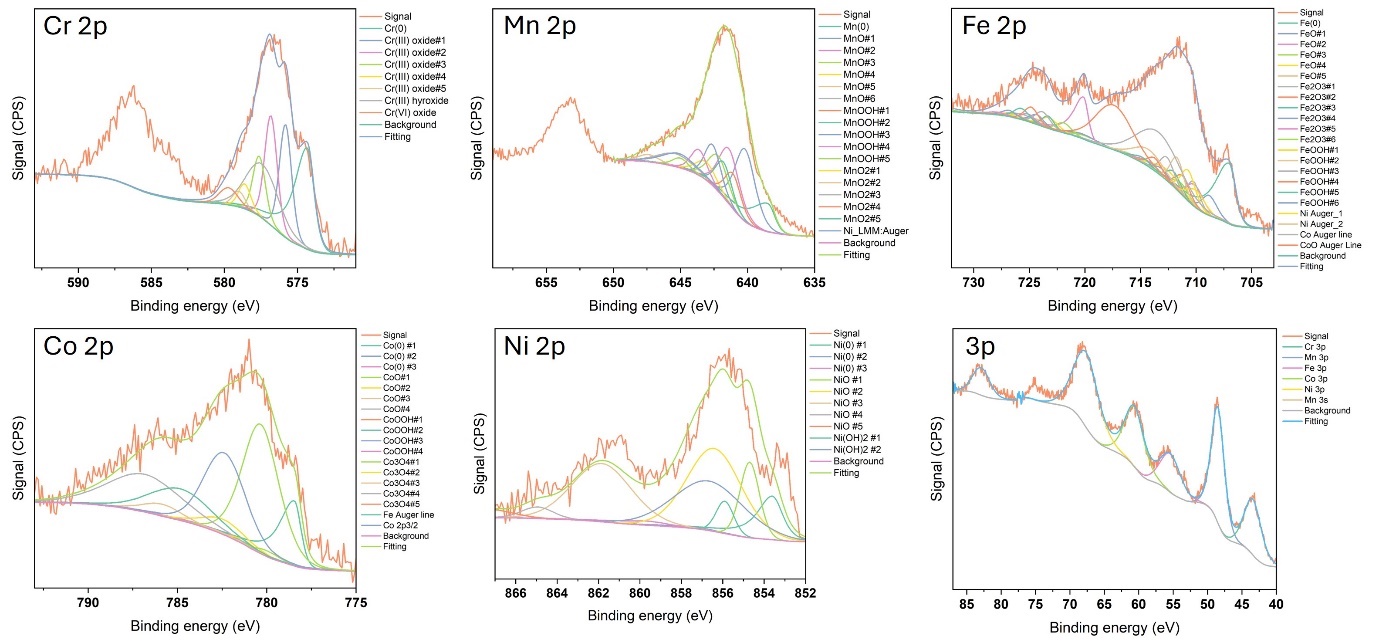


Figure S30 Deconvoluted Cr 2p, Mn 2p, Fe 2p, Co 2p, Ni 2p and 3p XPS spectra of HEA NPs synthesized in ethanol within the size set 50 nm < d < 20 nm.


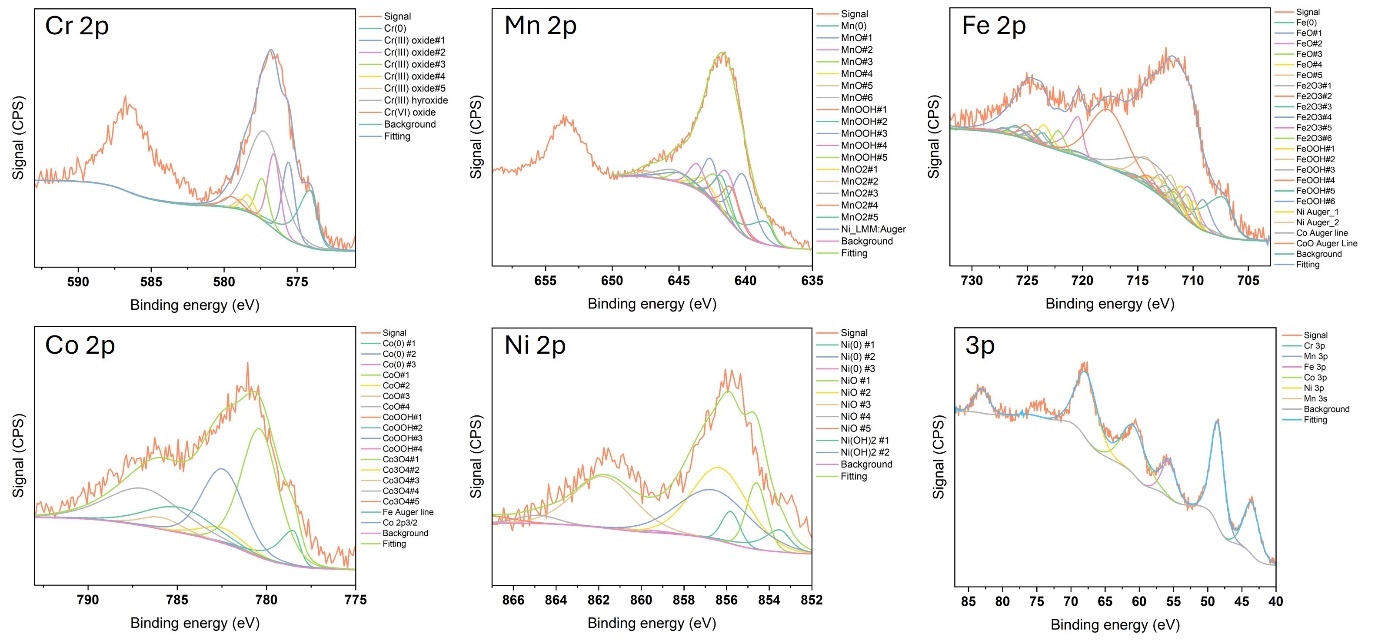


Figure S31 Deconvoluted Cr 2p, Mn 2p, Fe 2p, Co 2p, Ni 2p and 3p XPS spectra of HEA NPs synthesized in ethanol within the size set 20 nm < d < 10 nm.

**References**

[1] H.J. Jung, M.Y. Choi, One-pot synthesis of graphitic and nitrogen-doped graphitic layers on nickel nanoparticles produced by pulsed laser ablation in liquid: Solvent as the carbon and nitrogen source, Appl. Surf. Sci. 457 (2018) 1050–1056. https://doi.org/10.1016/j.apsusc.2018.07.036.

[2] H.-R. Cheng, H.S. Kim, Synergistic Amorphous Ni Core–N-Doped Carbon Shell Nanoparticles for Efficient Bifunctional Water Splitting, ENERGY Environ. Mater. n/a (n.d.) e70103. https://doi.org/10.1002/eem2.70103.

[3] S.-H. Hong, J. Winter, Micro-Raman spectroscopy on a-C:H nanoparticles, J. Appl. Phys. 98 (2005) 124304. https://doi.org/10.1063/1.2142078.

[4] A.C. Ferrari, J. Robertson, Resonant Raman spectroscopy of disordered, amorphous, and diamondlike carbon, Phys. Rev. B 64 (2001) 075414. https://doi.org/10.1103/PhysRevB.64.075414.

[5] L. Bokobza, J.-L. Bruneel, M. Couzi, Raman Spectra of Carbon-Based Materials (from Graphite to Carbon Black) and of Some Silicone Composites, C 1 (2015) 77–94. https://doi.org/10.3390/c1010077.

[6] F. De Geuser, B. Gault, Metrology of small particles and solute clusters by atom probe tomography, Acta Mater. 188 (2020) 406–415. https://doi.org/10.1016/j.actamat.2020.02.023.

[7] B. Gault, B. Klaes, F.F. Morgado, C. Freysoldt, Y. Li, F.D. Geuser, L.T. Stephenson, F. Vurpillot, Reflections on the Spatial Performance of Atom Probe Tomography in the Analysis of Atomic Neighborhoods, Microsc. Microanal. 28 (2022) 1116–1126. https://doi.org/10.1017/S1431927621012952.
